# Quantifying the Proteolytic Release of Extracellular Matrix-Sequestered VEGF with a Computational Model

**DOI:** 10.1371/journal.pone.0011860

**Published:** 2010-07-29

**Authors:** Prakash Vempati, Feilim Mac Gabhann, Aleksander S. Popel

**Affiliations:** 1 Department of Biomedical Engineering, Johns Hopkins University School of Medicine, Baltimore, Maryland, United States of America; 2 Institute for Computational Medicine and Department of Biomedical Engineering, Johns Hopkins University, Baltimore, Maryland, United States of America; University of Tor Vergata, Italy

## Abstract

**Background:**

VEGF proteolysis by plasmin or matrix metalloproteinases (MMPs) is believed to play an important role in regulating vascular patterning *in vivo* by releasing VEGF from the extracellular matrix (ECM). However, a quantitative understanding of the kinetics of VEGF cleavage and the efficiency of cell-mediated VEGF release is currently lacking. To address these uncertainties, we develop a molecular-detailed quantitative model of VEGF proteolysis, used here in the context of an endothelial sprout.

**Methodology and Findings:**

To study a cell's ability to cleave VEGF, the model captures MMP secretion, VEGF-ECM binding, VEGF proteolysis from VEGF_165_ to VEGF_114_ (the expected MMP cleavage product of VEGF_165_) and VEGF receptor-mediated recapture. Using experimental data, we estimated the effective bimolecular rate constant of VEGF_165_ cleavage by plasmin to be 328 M^−1^s^−1^ at 25°C, which is relatively slow compared to typical MMP-ECM proteolysis reactions. While previous studies have implicated cellular proteolysis in growth factor processing, we show that single cells do not individually have the capacity to cleave VEGF to any appreciable extent (less than 0.1% conversion). In addition, we find that a tip cell's receptor system will not efficiently recapture the cleaved VEGF due to an inability of cleaved VEGF to associate with Neuropilin-1.

**Conclusions:**

Overall, VEGF_165_ cleavage *in vivo* is likely to be mediated by the combined effect of numerous cells, instead of behaving in a single-cell-directed, autocrine manner. We show that heparan sulfate proteoglycans (HSPGs) potentiate VEGF cleavage by increasing the VEGF clearance time in tissues. In addition, we find that the VEGF-HSPG complex is more sensitive to proteases than is soluble VEGF, which may imply its potential relevance in receptor signaling. Finally, according to our calculations, experimentally measured soluble protease levels are approximately two orders of magnitude lower than that needed to reconcile levels of VEGF cleavage seen in pathological situations.

## Introduction

The cytokine vascular endothelial growth factor A (VEGF) is a critical mediator of adult neovascularization. Inducing blood vessel growth can be beneficial in alleviating tissue ischemia and in synthetic graft acceptance; however, neovascularization is also responsible for supporting pathological processes such as tumor growth. Controlling the activity of VEGF is thus an area of significant interest.

VEGF activity and patterning in tissues is regulated by its binding to the extracellular matrix (ECM), which is determined both by alternate splicing of VEGF and by processing of VEGF and the ECM by proteases and heparinases, resulting in a range of vascular phenotypes [1]–[5]. Alternate splicing results in isoforms of various lengths, the most actively expressed in humans being VEGF_121_, VEGF_165_, and VEGF_189_. The longer VEGF isoforms contain basic residues encoded by exons 6 and/or 7 of the VEGF gene, which results in differential binding to VEGF receptors (VEGFRs), Neuropilin-1 (NRP1) [6], [7], and to various ECM molecules including collagen, fibronectin, fibrinogen, and foremost, glycosaminoglycans, found in heparan sulfate proteoglycans (HSPGs) [1], [8]–[12]. ECM binding may regulate VEGF-dependent vascular patterning by controlling VEGF diffusion and gradients through tissues [5], [13] and possibly by mediating solid-state binding to VEGFRs [3], [14]–[16].

Proteolytic release of VEGF, also referred to as VEGF release (as distinct from secretion of the unproteolyzed ligand by cells), can occur by cleavage of matrix-bound VEGF at its C-terminal domain or by cleavage of the ECM and results in a diffusible VEGF [1], [3], [8], [17], [18]. VEGF release is thought to increase the soluble VEGF concentration, potentiating the angiogenic switch and leading to neovascularization and tumor growth [1], [8], [18], [19], but in some cases, it impairs angiogenesis [3], [20], [21] and deters tumor progression [3].

VEGF cleavage can occur readily via the proteases plasmin, MMPs, and elastase [3], [6], [17], [20], [22]. The structural requirements for VEGF cleavage are not currently well understood. While human VEGF isoforms are susceptible to proteolysis by plasmin [3], [6], [14], [23], they do not seem to be susceptible to the MMPs [8], [22], [24]. On the other hand, murine VEGF, e.g. VEGF_164_, the murine form of VEGF_165_, displays susceptibility to the MMPs, the most potent being MMPs -3, -7, -9, -12, and -19 [3], [17]. HSPGs seem to protect VEGF against some proteases, e.g. MMP9, but not against others, e.g. MMP3 [3].

VEGF release occurring through the cleavage of the ECM can occur through proteoglycan core protein digestion by plasmin, elastase, or a subset of the MMPs (e.g. MMPs -3, -9, -13, but not MMP2) [1], [8], [9], [25], [26]; or through GAG cleavage by heparinases [9], [27]. In tissue engineering applications, VEGF variants can be covalently tethered to matrices such as fibrin and polyethylene glycol [14], [28] to be protected against rapid diffusive clearance and allow VEGF release in a cell-mediated, matrix-coupled fashion [29]. It is not currently known whether VEGF cleavage or ECM cleavage is the predominant mechanism of VEGF release. Lee et al. [3] show evidence for the former, both in tumor xenografts and in an *in vitro* endothelial cell spheroid model, while Hawinkels et al. [8] demonstrate that MMP9 cleaves HSPGs to mediate the VEGF release-dependent angiogenesis of colon tumor explants.

Much remains to be determined about the nature of VEGF proteolysis and cell-mediated release. For example, endothelial cells [3], [19], neutrophils [30], and macrophages [17], [30]–[32] all have been implicated as potential mediators of VEGF release, but when each cell type is important is not known. Thus the question of when VEGF release is an autocrine or paracrine process has not been answered. In addition, the extent of VEGF release is system-dependent. Lee et al. reported that in the serum of mice implanted with fibrosarcoma, over 80% of circulating VEGF is in a cleaved form [3], a value similar to that in retinal tissue of mice with oxygen-induced retinopathy [17]. A lower, but still significant value of ∼30% cleaved VEGF was detected in human ovarian tumor lysates [33]. In contrast, in an *in vitro* fibrin-based system, a significant release-dependent cellular response occurred without any detectable VEGF release [14]. We do not currently know the rate at which VEGF release or cleavage occurs in biology. Experiments treating growth factors with exogenous protease show that 20 min with 400 nM plasmin [2], [6], [34], 30 min with 20 µM elastase [35], or 24 h with ∼0.3 to 20 nM active MMP9 [1], [8] all carry significant proteolytic potential. However, *in vivo* protease levels in plasma and in pathological fluid samples are typically lower, between 100 pM–20 nM [36]–[41], while clearance rates for VEGF (after accounting for its proteolytic degradation) are very rapid, ∼1 h [42]. It is not currently known if these protease levels are sufficient to account for the >80% circulating cleaved VEGF [3].

The efficiency of VEGF cleavage and release may be tied to intense but tightly localized pericellular events (difficult to detect using standard experimental techniques lacking spatial resolution) [3], [19], [43]. Numerous studies have shown proteases to be tightly localized around cells, through cell-surface molecules such as α2(IV), CD44, or LRP [44], [45]. Tight cell-surface or pericellular localization is known to improve protease activation [46] and reduce inhibition [47], [48], and thus may increase proteolysis of the ECM and of soluble growth factors, e.g. transforming growth factor-β and VEGF [3], [45], [49]–[51]. Indeed, our computational model has previously demonstrated that cell-surface MT1-MMP plays an important role in sprout migration [50]. Proteases may be further localized in an individual cell to distinct cell-surface microdomains, e.g. focal adhesions, which may enhance their activity. However, other studies show that it is interstitial proteases and not cell-surface proteases that are important in VEGF release and gradient formation [19], [52].

Quantitative understanding of these processes is essential to determine their relative importance *in vivo*. For example, while it is generally assumed that matrix binding localizes a growth factor to the ECM, generating local deposits of VEGF [5], [53], growth factor-ECM complexes can rapidly dissociate (k_off_ = 0.01 s^−1^) [54] and are thus highly dynamic, a criterion which affects the cleavage of VEGF. A central goal in this study is to determine the extent to which VEGF release is a cell-directed and localized or autocrine process. Computational and mathematical models by other groups have suggested that autocrine signaling of growth factors is feasible with low cytokine diffusivity in biological matrices [55] and that interstitial flow can generate protease-induced autocrine VEGF gradients [19]. Experimentally, MMPs have been shown to modulate growth factor signaling at the single cell level by directing lateral inhibition during branching morphogenesis [56]. It is also often thought that a single cell has sufficient proteolytic capacity to alter the VEGF distribution [19], [53], [57]. Here, we study the capacities of an endothelial tip cell to cleave VEGF and to recapture released VEGF, as well as determine whether known protease levels can result in the VEGF conversion levels (the ratio of cleaved VEGF to total soluble VEGF) observed both *in vivo* and *in vitro*.

To clarify the process of VEGF release, we develop a molecular-detailed quantitative model of VEGF cleavage based on an endothelial tip cell cleaving and releasing nearby VEGF. This model extends our efforts to quantify the interactions between VEGF, VEGF receptors, and the MMP systems to mechanistically understand angiogenesis and drug treatments to reduce angiogenesis in pathological situations [50], [58]–[67]. Using experimental data, we estimate rate constants for the proteolytic cleavage of VEGF by plasmin and specify protease secretion rates by a tip cell. We characterize the extent of VEGF cleavage as well as the cellular and tissue determinants of proteolytic VEGF release. We simulate tip cell-mediated redistribution of VEGF in tissues and compare the results with experimental data [19], [53]. The model includes both VEGF-HSPG binding and the VEGF-VEGFR interactions to simulate autocrine capture. The present model is specifically applied to the geometry of a protease-secreting endothelial tip cell, however our model is extendable to any cell type.

## Methods

### Model Formulation

**Figure 1:**
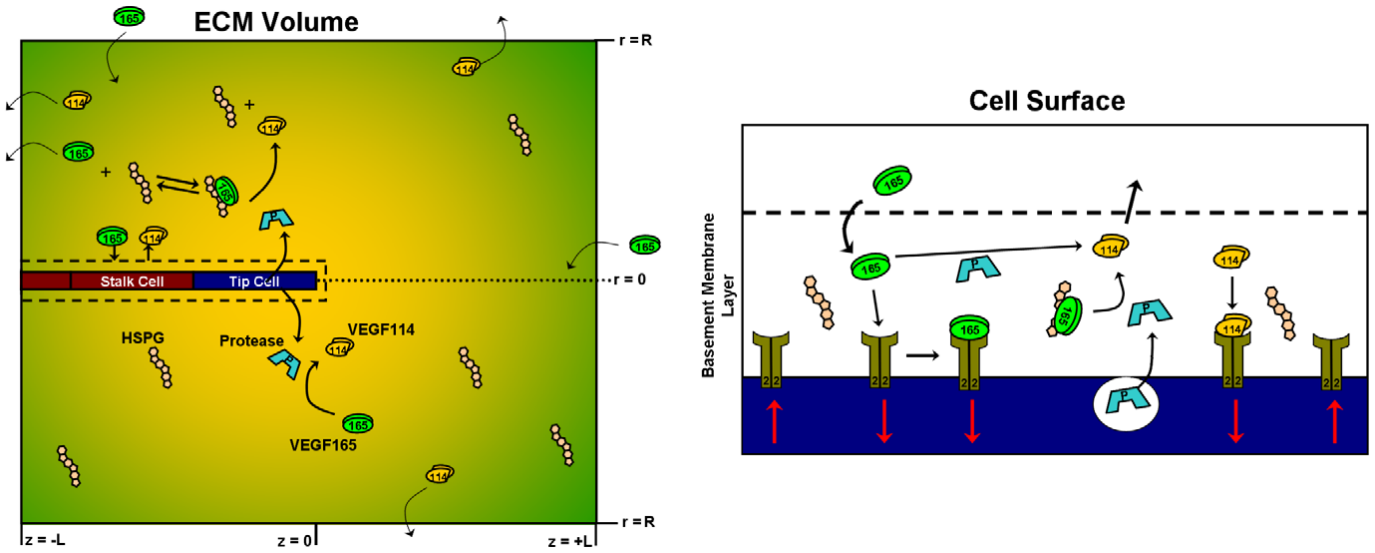
Schematic of computational model. VEGF is represented as either VEGF_165_ (green) or VEGF_114_ (orange), and secreted protease as P (light blue). VEGF_165_ is able to diffuse and bind HSPG, bind to VEGF receptors, or become proteolyzed by proteases secreted by the tip cell of the endothelial sprout. Computationally, the sprout is located in the ECM volume but is surrounded by a narrow basement membrane. The matrix molecule of importance in the present model is the HSPG, which is able to reversibly bind VEGF. Protease is assumed to catalyze the conversion of VEGF_165_ to VEGF_114_ in a one-step reaction (*see text*). For simplicity, our model considers only a single VEGF receptor, VEGFR2.

We developed a computational model to calculate the proteolytic cleavage of VEGF by a protease-secreting endothelial sprout (Fig. 1). We consider a region of tissue actively undergoing angiogenesis with numerous sprouts projecting towards a distant VEGF source, e.g. a tumor; our model system focuses on one of those sprouts, to capture the behavior of proteases and VEGF around it. We included only one VEGF isoform, VEGF_165_; its transport includes diffusion, protease interactions, and binding to HSPGs and VEGFRs (which mediate cellular internalization of VEGF). The sprout, idealized as a cylinder, consists of a single tip cell followed by stalk cells, is surrounded by a thin basement membrane (BM), and is immersed in a volume of ECM. We assume that the endothelial tip cell is “activated” and can secrete a generic protease capable of cleaving both free and ECM-bound VEGF_165_ into VEGF_114_. Along with diffusion of protease, VEGF_165_, and VEGF_114_, the model simulates the following reactions:

### Volumetric Reactions (ECM and BM)

VEGF_165_ + HSPG ↔ VEGF_165_·HSPG

VEGF_165_ + Protease → VEGF_114_ + Protease

VEGF_165_·HSPG + Protease → VEGF_114_ + Protease + HSPG

### Cell-surface (Heterogeneous) Reactions (BM only)

VEGF_165_ + VEGFR2 ↔ VEGF_165_·VEGFR2

VEGF_114_ + VEGFR2 ↔ VEGF_114_·VEGFR2

The three reversible reactions above are characterized by kinetic rate constants for association and dissociation. VEGF_165_ binding to HSPGs is modeled as a single-step reaction [54]. While VEGF_165_ cleavage to VEGF_114_ is in reality a two-step reaction [6] occurring through the intermediate heterodimer, VEGF_165-114_, we assume an effective single cleavage step (justified in *Supplement S1*, *section S1*; *Fig. S1B*). VEGFR2 is inserted into and internalized from the cell surface. Protease is secreted into the basement membrane layer and diffuses out into the ECM volume (Fig. 1). For simplicity, we assume that the secreted protease is active, i.e. we ignore the dynamics of its activation and inhibition, which we have previously characterized for MMP9 and MMP2 [50], [58], [59]. In this model, we are primarily interested in characterizing the extent of VEGF cleavage at steady-state. Our calculations proceed in two steps: first to calculate the VEGF distribution before the secretion of proteases and then incorporate protease secretion and VEGF proteolysis until a final steady state is reached.

For simplicity our current model does not incorporate VEGFR1 or NRP1, receptors for which we have previously developed biochemically-detailed models [62], as we assume receptor binding primarily plays a sensory role and the model instead focuses on characterizing VEGF proteolysis. We note that one of our results is concerned with the autocrine capture of VEGF, for which we also test the role of VEGFR1 and NRP1 and thus provide a full reaction formulation for reference in *Supplement S1*, *section SII*, which follows [68]. Similarly, we also test whether NRP1 affects the assumption of VEGF proteolysis occurring in a single step (*Supplement S1*, *section SIII*; *Fig. S2*).

The basement membrane covering vessels may not always be structurally integral, e.g. in tumors [69]. In addition, for a nascent sprout, basement membrane deposition by the sprout lags the sprout's migration resulting in basement membrane coverage over the entire sprout except for the tip cell, as visualized in [70]. In our model, we assumed a complete basement membrane over the entire sprout as a reference case; we also tested the effects of varying basement membrane thickness and partial tip cell basement membrane coverage and found the effect of basement membrane coverage to be negligible. The ECM and basement membrane were treated as porous media due to the presence of fibrillar components. The porous material property of interest, the available volume fraction, K_av_, represents the fraction of space that is accessible to a molecule for diffusion and depends on the molecule's size and matrix fiber composition [71], [72]. As a result, the concentration of a molecular species in the interstitial fluid, C_fluid_, or “local” concentration, differs from the volume-averaged “bulk” concentration, C_bulk_, of the species in a tissue by the relation C_bulk_ = C_fluid_·K_av_. The ECM and basement membrane are physically distinct, which is reflected in our model by different values for the protein diffusivities, K_av_, HSPG density, and reactive components (see *Methods*, *Parameters*). While our governing equations are formulated in terms of bulk concentrations, we will refer to a species' interstitial fluid concentration in our results and figures unless explicitly stated.

### VEGF/Protease Transport and Reactions in the ECM

Our system is formulated mathematically as reaction-diffusion partial differential equations:
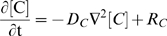
(1)where D_C_ is the effective diffusivity of a molecular species C in the porous environment and [C] is the bulk or pore-averaged concentration of C. For each species, these differential equations (Eqns. 2–6) are coupled to their appropriate boundary conditions (see *Methods* *, External Boundary Conditions* and *Methods* *, Reactions at Vessel Boundary*).

We first consider the transport of VEGF_165_ in the domain:
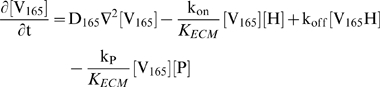
(2)The reaction terms, k_on_ and k_off_, reflect the binding of VEGF_165_ to the HSPG, while k_P_ denotes the effective bimolecular rate constant of proteolysis between VEGF_165_ and protease, given by [P].

VEGF_114_ is generated from cleavage of either free or matrix-bound VEGF_165_ [3]:
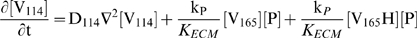
(3)A C-terminal fragment of VEGF is also produced from cleavage by plasmin and while it has affinity to the matrix [6], its effects can be neglected as its concentration is much less ([V]∼1 pM [73]) than the K_d_ of matrix interaction, and thus does not saturate existing binding sites.

Our estimated K_m_ of VEGF proteolysis by plasmin (>1 mM, see *Supplement S1, section SI*) is much larger than the VEGF concentration and allows us to use first-order, rather than Michaelis-Menten, kinetics for the cleavage and neglect changes in the protease concentration due to VEGF binding. To account for possible protease inactivation due to inhibition or degradation, we impose an autolytic degradation rate constant, k_deg_. Thus, the protease distribution after its secretion is governed by:
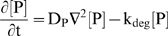
(4)


The matrix components, HSPG ([H]) and VEGF_165_-HSPG ([V_165_H]), are described as ordinary differential equations (ODEs) since they do not diffuse but do interact with VEGF and protease:
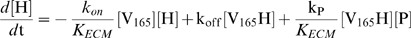
(5)

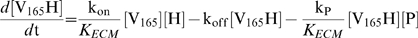
(6)


### External boundary conditions for VEGF secretion and interstitial clearance

Typically, boundary conditions are imposed by specifying either the concentration or the flux at each face. We assume a cylindrical coordinate system and thus must specify four external boundary conditions: the leading face, z = +L; trailing face, z = −L; radial edge, r = R; and the central axis in the region excluding the sprout, i.e. r = 0 and z>0 (see Fig. 1). The vessel surface boundary is considered in the next section.

For the central axis (r = 0), we have ∂V/∂r = 0 due to symmetry. We consider two cases of the model:

Case 1: The sprout is considered in isolation, with an imposed background of VEGF or protease. Thus we use a Dirichlet boundary condition: [V_165_] = V_0_, [V_114_] = 0, and [P] = P_0_ at all outer boundaries, r = R and z = ±L.

Case 2: The sprout is considered as one of many sprouts migrating up an imposed gradient of VEGF. The computational domain is assumed representative of its surrounding tissue in the radial direction. Thus, we use a no-flux condition, ∂V/∂r = 0 at r = R. With the VEGF gradient in the z-direction, Dirichlet boundary conditions would overestimate VEGF diffusion through the boundaries (τ_Diffusion_∼L^2^/D∼1.5 min) compared to *in vivo* clearance rates on the order of hours [42]. Thus, Neumann boundary conditions were used to specify the VEGF_165_ secretion rate from the surface z = +L: −D·∂[V_165_]/∂z = −q, which was balanced by a first-order VEGF clearance at z = −L, −D·∂[V_165_]/∂z = −k_clear_[V_165_]. The secretion rate q and k_clear_ were pre-calculated to give the desired VEGF concentration in the absence of proteases, V_0_ at z = 0 and VEGF gradient, g_0_ over the domain length (see *Methods*, *Model Implementation*). For VEGF_114_, ∂[V_114_]/∂z = 0 at z = +L (no secretion), and −D·∂[V_114_]/∂z = −k_clear_[V_114_] at z = −L. The overall clearance rate, k_clear_/(2·L) ∼5.41·10^−4^ s^−1^, is similar in magnitude to clearance times *in vivo* [42] and represents receptor-mediated internalization by the pre-existing vasculature and transvascular permeability. For simplicity, we assume a uniform protease distribution. While protease patterning would affect the resulting VEGF distribution, we are mainly interested in the degree of VEGF cleavage brought about by the mean protease level, and not in the specific shape of VEGF gradients, which may depend on which cells secrete proteases and where.

### Reactions at Vessel Boundary and Transport in the Basement Membrane

Basement membranes around vessels are thin (∼43 nm [74]). Thus, the diffusive hindrance over the basement membrane layer's length is formulated as a lumped boundary condition such that at the transition between the ECM and basement membrane layer, both the interstitial fluid concentrations of solutes and the total diffusive fluxes (given by J_out_) are continuous (refer to *Supplement S1, section SIV*):
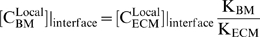
(7)

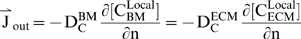
(8)


Cell-surface reactions are assumed to occur in the basement membrane volume and lateral diffusion along the cell surface is negligible (D∼10^−2^ µm^2^/s [75]; Damkohler number, *Da* = k_off_·L_sprout_^2^/D = 160). As a result, the cell-surface distributions of soluble species and receptors can be approximated by ordinary differential equations (Eqns. 9–16)
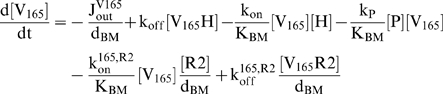
(9)

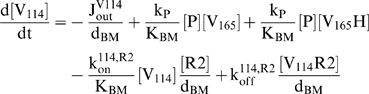
(10)

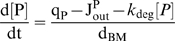
(11)


We assume constant total HSPG concentration, with no secretion by the endothelial cell. Similar to the case in the ECM, we have:
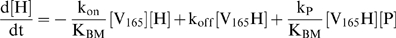
(12)

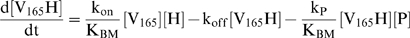
(13)


Receptor and ligand-receptor complex distributions are also assumed to be homogenous over the cell surface, i.e. we do not include the effects of receptor clustering [76]. VEGFR2 is under a constant flux of cell-surface expression (insertion) and internalization, which we assume is independent of its binding VEGF.
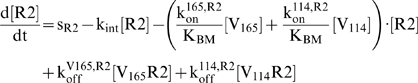
(14)

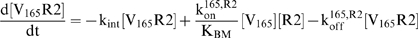
(15)

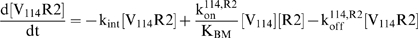
(16)


It has been shown that VEGF_110_ (or VEGF_114_ in our case) behaves similarly to VEGF_121_ [6], and thus its interactions with VEGFR2 follow the same formulation as VEGF_165_ or VEGF_121_. In the current model, we assume that only free VEGF can interact with VEGF receptors. That is, VEGF_165_ bound to HSPG in the basement membrane layer cannot interact with the receptor population. The kinetic parameters describing these reactions are described below (see Tables 1, 2). We will make inferences about the possibility of matrix-sequestered VEGF species interacting with VEGF surface receptors in the Discussion.

**Table 1:** Parameters of Model.

Definition	Parameter	Value	Source
***Kinetic Parameters***			
Association with VEGFR2	k_on_^114,R2^, k_on_^165,R2^	1·10^7^ M^−1^s^−1^	[61]
Dissociation with VEGFR2	k_off_^114,R2^, k_off_^165,R2^	1·10^−3^ s^−1^ (K_d_ = 100 pM)	[61]
Internalization rate of VEGFR2	k_int_	2.8·10^−4^ s^−1^	[79]
Expression rate of VEGFR2	s_R2_	k_int_·[R2]_Total_	†
Association with HSPG	k_on_^165,H^	4.2·10^5^ M^−1^s^−1^	[54]
Dissociation with HSPG	k_off_^165,H^	0.01 M^−1^s^−1^ (K_d_ = 24 nM)	[54]
Cleavage rate of VEGF by Protease	k_P_	631 M^−1^s^−1^, see *Results*	[6], [95] ‡
Secretion rate of Protease from tip cell	q_P_	3·10^6^ molecules/h (2.7·10^−9^ [mol/(10^15^ µm^2^·s)]	[86]
***Transport and Physical Parameters***			
Length of tip cell	L_tip_	40 µm	[13] ‡
Radius of sprout	R_sprout_	2 µm	[13] ‡
Area of tip cell surface	A_tip_	515 µm^2^	†
Basement membrane thickness	d_BM_	0.043 µm	[96]
Available volume fraction of ECM	K_ECM_	0.85	[71] ‡
Available volume fraction of BM	K_BM_	0.20	[71] ‡
Diffusivity	D_165_, D_114_, D_P_	68.8 µm^2^/s (ECM), 18.0 µm^2^/s (BM)	† see *Methods*

† Calculated.

‡ Estimated from Ref.

**Table 2:** Base Conditions.

Definition	Parameter	Value	Source
*Common Conditions*			
Total interstitial-fluid [HSPG] ECM	[H_ECM_]_Total_	0.75·10^−6^ mol/(10^15^ µm^3^)	[97]
Total interstitial-fluid [HSPG] BM	[H_BM_]_Total_	13·10^−6^ mol/(10^15^ µm^3^)	[54]
Total VEGFR2 per cell	[R2]_Total_	10,000/(area of tip cell) -i.e.- 3.22·10^−8^ [mol/10^15^ µm^2^]	[61]
*Case 1 (isolated cell)*			
Farfield [VEGF_165_]	V_0_ (farfield)	1 pM	[73]
Farfield [Protease]	P_0_ (farfield)	0 nM	*
Length, Radius of cylindrical domain	L, R_edge_	1200 µm, 1200 µm	*
*Case 2 (non-isolated cell)*			
Initial mean [VEGF_165_]	V_0_	1 pM	*
VEGF secretion rate on surface, z = +L	q_V_ (z = +L)	7.68·10^−5^ molec/µm^2^·s	†
Fractional gradient at z = 0	g_0_	0.05	*
Clearance rate on surface, z = −L	k_clear_	0.0866 µm/s	*
Length, Radius of cylindrical domain	L, R_edge_	80 µm, 50 µm	*

†Calculated.

*Assumed.

### Numerical Methods for VEGF Calculations

The computational domain was represented in cylindrical coordinates and the VEGF and HSPG transport equations were solved using the finite volume method on a 2D grid (z and r). The basement membrane layer was used to approximate cell-surface and basement membrane volume reactions. The control volume spacing in the z-direction was 8 µm. In the r-direction, one voxel was used to represent the sprout radius, from r = 0 to r = R_sprout_; for r≥r_sprout_ (outside the sprout surface), spacing was 4–8 µm. Due to the thinness of the basement membrane layer, its radial dimension was approximated by a single node. J_out_, previously given implicitly, was derived such that fluxes and concentrations were continuous (refer to *Supplement S1*, *section SIV*).

The first order derivatives in time were discretized using a first order fully-implicit scheme, while second order spatial derivatives used a central difference approximation. Nonlinear solution of the equations was found by iteration using the successive over-relaxation (SOR) update formulation and a Red-Black node ordering [77]. Additional speedup was performed by setting the initial guess for the solution of each time step as y^t+1^ = 2·y^t^−y^t−1^. The convergence criteria at each time step was set to a maximum fractional change in any computational node less than or equal to 10^−7^/iteration.

### Model Implementation and Initial Conditions

The initial condition for the protease secretion simulations consists of a fully-formed VEGF distribution after taking into account VEGF depletion by receptor-mediated internalization. For case 1, this consisted of finding the steady-state distribution in the absence of protease secretion. In the case of an imposed VEGF gradient (case 2), we used Neumann boundary conditions fitted to produce a mean concentration, V_0_, and gradient, g_0_, and thus required a sequence of simulation steps.

We first specified Dirichlet boundary conditions in the absence of receptors to satisfy g_0_ (see Parameters) and the mean VEGF concentration at z = 0, V_0_: at z = +L, [V_165_] = V_0_·(1+g_0_·L/L_tip_), at z = −L, [V_165_] = V_0_·(1−g_0_·L/L_tip_)). This was done in the absence of HSPGs to allow faster convergence. After equilibration of the VEGF receptor population, the Dirichlet boundary conditions were converted into the appropriate ‘case 2’ boundary conditions by calculating q and k_clear_ using the following formulas,
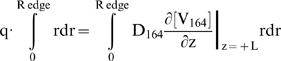
(17)

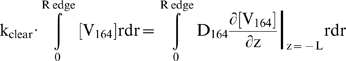
(18)and the system was solved until steady state was reached. Even though these boundary conditions do not exactly specify the conditions g_0_ and V_0_, this approximation is justified since the error is small for our domain size and gradient. HSPGs were finally directly imposed by the equilibrium relations [V_165_H] = [V]·[H]_Total_/(K_d_+[V]), as the distribution of soluble VEGF at steady state is independent of HSPGs in the absence of proteases.

### Geometrical and Transport Parameters

The domain sizes were L = R_edge_ = 1200 µm (case 1) and L = 80 µm and R_edge_ = 50 µm (case 2) (Fig. 1). The case 1 dimensions are large to study the impact of a single sprout in isolation. The case 2 domain dimensions are representative of mean sprout to sprout distance observed in retinal angiogenesis studies (50–100 µm) [13] and mean neutrophil to neutrophil distance in pancreatic islets (i.e. given a neutrophil frequency of 0.1% to 0.4%, neutrophil to neutrophil distance ∼200 µm) [30], as well as typical sprout tip cell lengths of 40 µm. In these domains, the sprout is idealized as a cylinder from the surface z = −L to z = 0 of which the tip cell occupies from z = −40 µm to z = 0 µm. The radius of the sprout cylinder is 2 µm [13].

The diffusivity and available volume fractions depend on the structure of the interstitial matrix. We estimated diffusivities directly from the properties of the ECM (collagen: v/v = 14%, fiber radius = 20 nm; glycosaminoglycans: v/v = 0.078%, effective fiber radius = 0.55 nm [78]) and estimates of interstitial protein content (*Supplement S1*, *section SV*; *Table S2*). While the diffusivities of various VEGF isoforms would be different *in vivo*, we assumed each had a diffusivity equal to that of VEGF_165_. Available volume fractions were taken as limiting cases for matrix connectivities as derived in [71] (K_ECM_ = 0.85, K_BM_ = 0.2).

### Kinetic Parameters of Reactions

The kinetic parameters for VEGF binding to VEGFR2 have been previously characterized [61], while VEGF_165_ binding to HSPGs was assumed to be identical to that of bFGF [54]. To characterize the kinetic rate constants of VEGF cleavage by proteases, we assumed the following molecular weights: active plasmin (86 kDa with commercial preparations containing 3 U/mg); MMP3 and VEGF_165_, 45 kDa each. Plasmin and MMP3 seemed to have similar proteolytic strengths on a molar basis (refer to supplement of [3]). One study indicated VEGF_165_ cleavage by plasmin (0.01 U/mL or ∼40 nM) from zymographic and VEGF mitogenicity data with a half-life of 1–4 h, equating to k_P_ ∼1.2·10^3^–5·10^3^ M^−1^s^−1^ [34]. Our estimate of the rate of VEGF_165_ cleavage by plasmin at 37°C (k_p_ = 631 M^−1^s^−1^) was derived by directly fitting kinetic data from Keyt et al. [6] to a one-step cleavage model (described in *Supplement S1*, *section S1*; *Fig. S1A*).

### Concentrations of VEGF, HSPG, Receptors and Proteases

Interstitial VEGF levels were previously reported at ∼1 pM [73], while HSPG concentrations were taken from previous studies at 750 nM in the ECM and 13 µM in the basement membranes [61]. We assume that these concentrations refer to intra-pore concentrations. Typical concentrations of VEGFR2 on abluminal faces of endothelium were estimated in previous studies at ∼10^4^ VEGFR2/cell [79], [80].

The level of active proteases in *in vitro* and *in vivo* biological systems is not exactly known. MMP and plasmin concentrations in the circulation have been determined previously to be <20 nM [81], [82]. The concentrations of the tissue inhibitors of metalloproteinases (TIMPs) have been also determined to be in the range of 1–10 nM [83]. Yao et al. have determined the concentration of active MMP2 in a fibroblast cell culture to be 100–350 ng/mL (1.6–5.6 nM) [11]. However, the distribution and cellular localization of this protease is not known.

The rate of MMP secretion is also a critical parameter affecting MMP localization and activity, and depends on cell type and factors used for cell stimulation. However as most cells, with the exception of neutrophils, secrete TIMPs in conjunction with proteases, the protease secretion rate itself is of limited use. For example, rabbit brain capillary endothelial cells secrete as much MMP as rabbit synovial fibroblasts, however the MMPs remain inactive, unlike in the latter, even after activators are present [84]. TIMP secretion is in the range of 1.5·10^5^–2·10^5^ molecules/cell/h [85], whereas MMP secretion has been found to range from ∼6·10^4^–10^7^ molecules/cell/h [84], [86]–[89]. In addition, without continuous protease expression, protease secretion may be a self-limiting process. The present model uses a simplification of the MMP dynamics, not taking into account protease synthesis, activation, inhibition, or internalization. We assume a constitutive protease expression of 3·10^6^ active protease molecules/cell/h. We expect that this serves as an upper bound to the real value of protease activity.

## Results

### A single endothelial tip cell causes little conversion of soluble VEGF

An analysis of kinetic data of cleavage of VEGF_165_ by plasmin yielded a bimolecular rate constant of k_P_ = 631 M^−1^s^−1^ at 37°C, which is at the low end of typical ECM enzyme-substrate reactions for the MMPs (refer to *Supplement S1*, *section S1*; *Table S1*). With low nM levels of MMPs *in vivo*, cleavage of VEGF would seem negligible as its time constant ∼100 h. These results motivate us to study the possibility of pericellular localization of MMPs upon their secretion.

**Figure 2:**
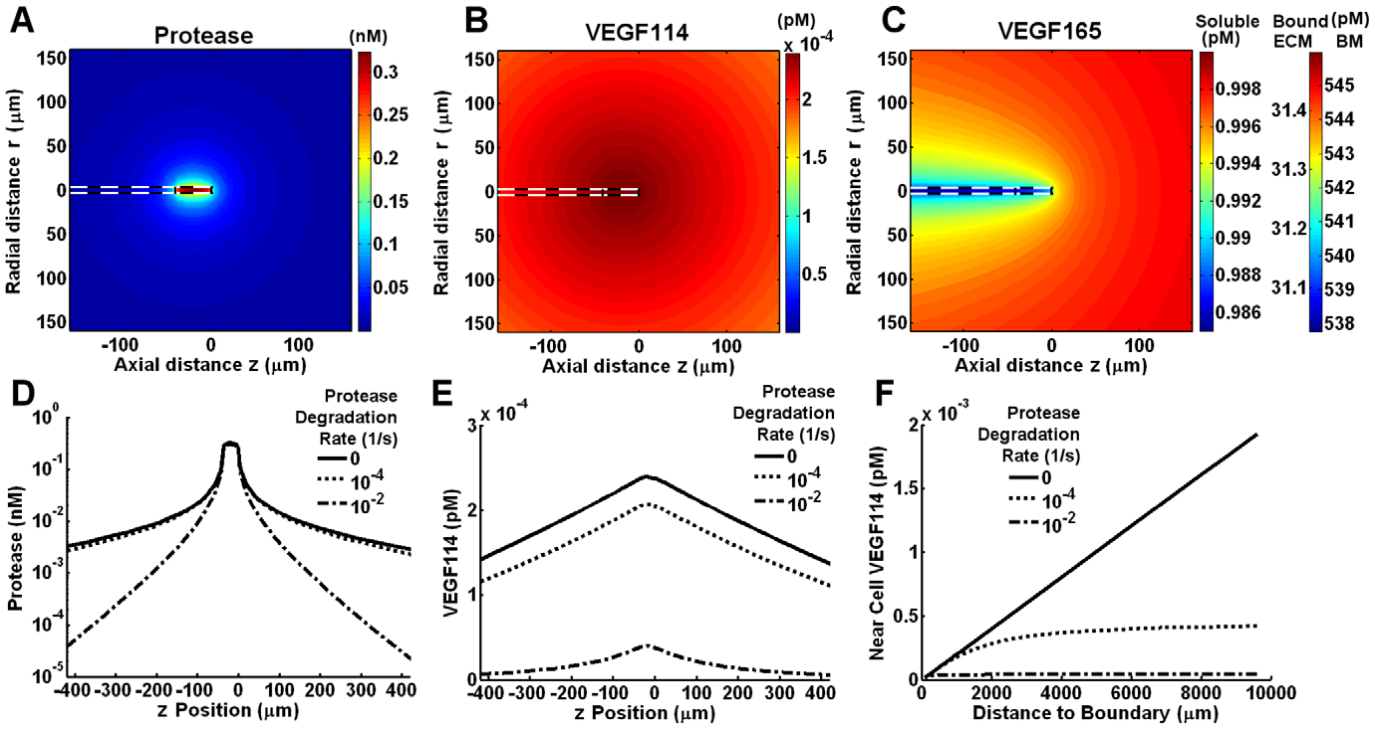
VEGF cleavage due to protease secretion from the tip cell of a vessel sprout. We considered the proteolysis of free and bound VEGF_165_ to VEGF_114_ by secretion of active protease uniformly from the tip cell surface at a rate of 3·10^6^ molecules/h. The distributions of protease, VEGF_114_, and VEGF_165_ are shown in A–C. We note that the VEGF_165_ distribution is virtually unaltered by the secreted proteases. Effects of degradation of protease activity (k_deg_ = 0, 10^−4^, or 10^−2^ s^−1^) on pericellular protease and VEGF_114_ (D–F). F, effect of boundary placement on pericellular VEGF_114_.

We considered an isolated tip cell secreting 3·10^6^ active protease molecules/h in a 3D reaction-diffusion model (Fig. 2). As a reference, we considered the sprout to be immersed in an initial VEGF concentration field of 1 pM. Note that VEGF levels are depleted to ∼0.988 pM at the tip cell due to internalization from the 10^4^ VEGFR2/cell along the sprout length (Fig. 2C).

Assuming that the secreted proteases are not inhibited or inactivated, a single cell secreting proteases in isolation provides a very low proteolytic load (∼0.31 nM in the available pores) at the secreting cell's surface (Fig. 2A, D). Additionally, due to the intrinsically slow kinetics of VEGF proteolysis, [VEGF_114_] is present at negligible levels, 2.4·10^−4^ pM (Fig. 2B, E), relative to the initial soluble VEGF. As a result, the VEGF_165_ distribution is barely altered by the cell's secretion of proteases (Fig. 2C). The total soluble VEGF distribution (VEGF_165_+VEGF_114_) is not affected by proteases.

The size of the volume around the sprout impacts VEGF conversion. Increasing the space around the single tip cell increases the total VEGF_114_ levels found in its vicinity (Fig. 2F) by increasing the total amount of protease a VEGF_165_ molecule originating at the boundary must diffuse through (in the limit of an infinite domain, there would be 100% conversion). Imposing protease degradation to mimic the effects of inhibition (as performed in [19], [53]) significantly decreases pericellular VEGF_114_ levels (Fig. 2 D–F).

**Figure 3:**
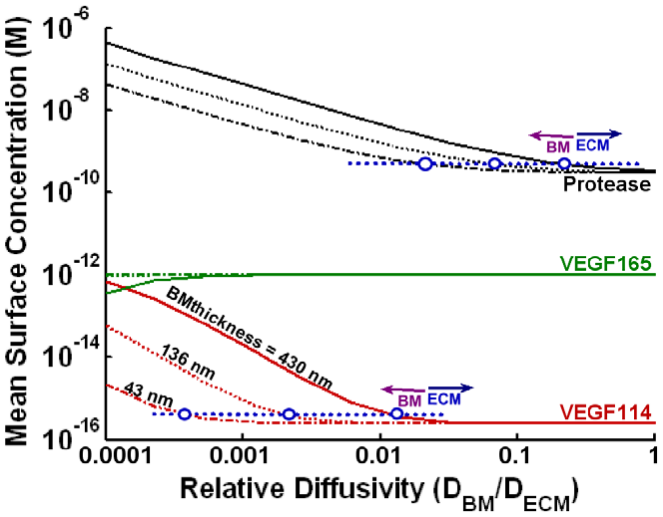
Effects of basement membrane thickness and diffusivity on cell-surface VEGF proteolysis. We studied the role of basement membrane (BM) diffusion on protease accumulation and VEGF conversion, in the absence of VEGFR2 and at domain size of 1200 µm. VEGF conversion at the cell surface does not reach appreciable levels unless D_BM_ is made significantly smaller than D_ECM_ (at 10^−4^-fold). A ten-fold decrease in diffusivity is equivalent to a ten-fold increase in basement membrane thickness. We note that the sum of VEGF_165_ and VEGF_114_ is a constant 1 pM at all conditions. The conditions for which basement membrane dynamics dominates the cell-surface concentration of VEGF are demarcated roughly by the intersection points of the dotted blue lines with the VEGF_114_ and protease curves.

While protease levels reached only 0.3 nM at the cell surface in our model, hindering basement membrane diffusion by increasing its thickness or decreasing the diffusivity may be able to increase pericellular protease levels and subsequently increase VEGF conversion. We find that pericellular protease levels are relatively sensitive to the basement membrane thickness and diffusivity (noticeable changes in its concentration begin at D_BM_/D_ECM_∼0.1). However, as the basement membrane diffusivity decreases, the increase in protease levels does not lead to a concomitant increase in VEGF cleavage, shown by cleaved VEGF not becoming significant until the basement membrane diffusivity reached 1/10,000^th^ of the ECM diffusivity (∼0.01 µm^2^/s) (Fig. 3). Thus, our model shows that the shape of the basement membrane has negligible effects on overall VEGF transport for physiologically realistic basement membrane properties (Table 1). Removing the basement membrane over the entire tip cell decreased MMP levels by ∼5% but decreased VEGF conversion by only ∼0.04% (*not shown*).

Further localization of MMP activity, e.g. to specific microdomains on the tip cell, may also increase local MMP concentrations and VEGF cleavage. To test this, we concentrated the entire MMP secretion to the leading edge of the tip cell. Similar to the above trends, increases in local MMP levels were substantial (to ∼5.5 nM) while increases in cleaved VEGF were not (*not shown*).

### Cell-surface proteases can deplete matrix-bound but not soluble VEGF

**Figure 4:**
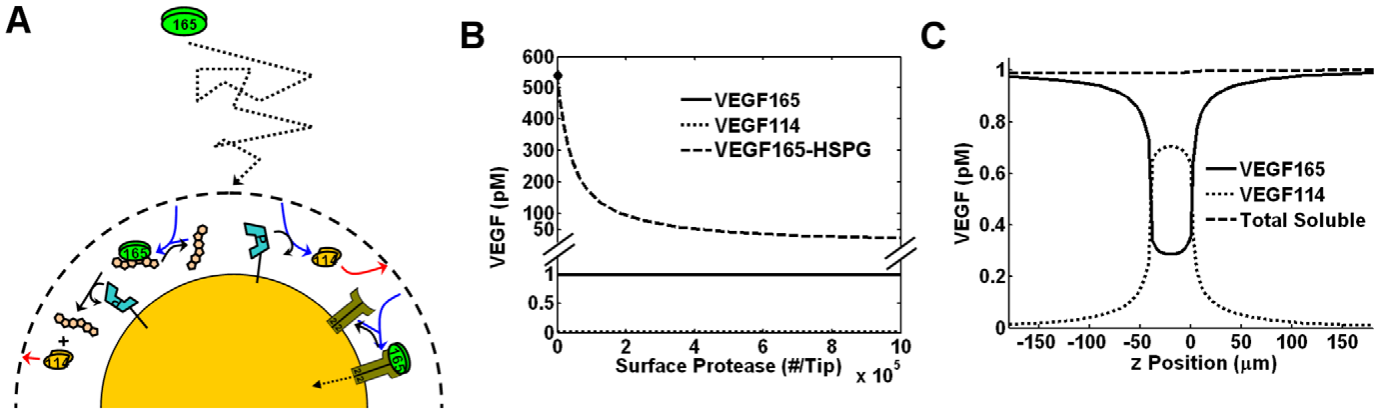
Cell-surface mediated VEGF proteolysis. To test VEGF_165_ conversion via cell-surface proteases, we restricted a specific number of proteases to the basement membrane layer of the tip cell (A). VEGF_165_ cleavage can occur either via direct encounter with a protease or after an initial complexation with HSPG, both at k_P_ = 631 M^−1^s^−1^. At 10^5^ proteases affixed to the tip cell, pericellular VEGF_114_ became ∼3.6·10^−3^ pM while VEGF_165_ decreased by the same amount; in contrast, VEGF_165_-HSPG in the basement membrane layer decreased from 540 pM to 160 pM (*not shown*). B, range of cell-surface protease densities. Note lack of VEGF_165_ conversion. C, pericellular VEGF distribution for 10^10^ cell-surface proteases (significantly higher than physiological levels). Note that total soluble VEGF (dashed line) is unaltered, even as VEGF_165_ undergoes significant depletion.

Cell-surface association may potentiate the strength of proteases against VEGF. To test this, we considered proteases now tethered to the cell surface that can react with VEGF_165_ and VEGF_165_-HSPG present in the basement membrane layer (Fig. 4A). This proteolysis mechanism would likely also apply to VEGF bound to cell-surface HSPGs, which is a more relevant scenario for a tip cell lacking a basement membrane.

As a baseline, we consider the tip cell having 10^5^ active cell-surface proteases [50]. With an initial background soluble [VEGF_165_] of 1 pM, proteolysis leads to the formation of only 3.6·10^−3^ pM cleaved VEGF at the cell surface (*not shown*). VEGF_165_ levels in the basement membrane dropped by an identical amount, indicating that VEGF_165_ levels in the ECM, and thus VEGF_165_-HSPG levels in the ECM, are not significantly altered (*not shown*). Unexpectedly, we notice a very sharp depletion of basement membrane VEGF_165_-HSPG (from 540 pM to 160 pM) (*not shown*).

The sensitivity of VEGF_165_-HSPG in the basement membrane to cell-surface proteases is more poignantly captured by testing a range of protease concentrations (Fig. 4B). The basement membrane soluble VEGF fractions do not noticeably change over this range, with their conversion only being significant when the tip cell expressed ∼10^10^ cell-surface proteases (Fig. 4C) (VEGF_165_ conversion = 69.5%, VEGF_165_-HSPG conversion = 100%), an unphysiological level.

To understand why basement membrane VEGF_165_-HSPG depletion occurs despite any noticeable depletion in VEGF_165_ itself, we turned to an analytical analysis of VEGF_165_ diffusion, binding, and proteolysis at the cell surface (refer to *Supplement S1*, *section SVI.1*; *Fig. S3*). We find that the difference in the susceptibilities of VEGF_165_ and VEGF_165_-HSPG to cell surface proteolysis stems from differences in their rates, per molecule basis, of being replenished after proteolysis. While VEGF_165_ is quickly replenished by diffusion (at a rate 1059 s^−1^), VEGF_165_-HSPG is replenished only as quickly as another complex can dissociate (i.e. k_off_ = 0.01 s^−1^). In our model, the rate of proteolysis per molecule VEGF is k_P_·[P] = 0.0236 s^−1^ ([P] is the effective concentration of 10^5^ proteases/cell in the available pores, or 37.5 µM).

### Extracellular protease accumulation can account for the proteolysis of VEGF

**Figure 5:**
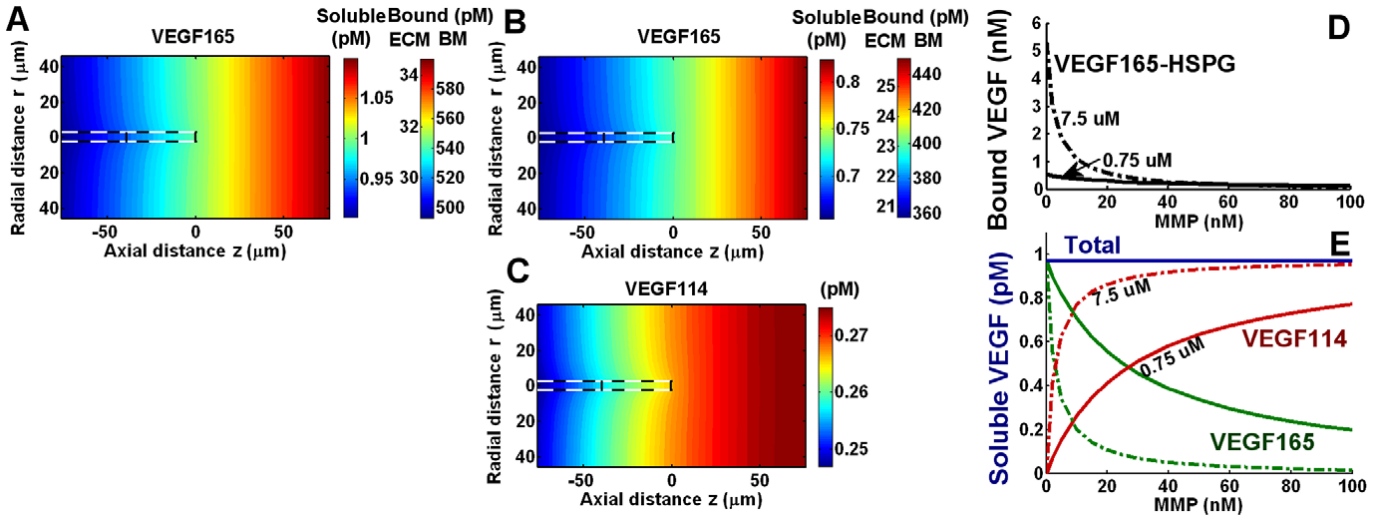
VEGF cleavage in a spatially bounded domain with VEGF gradient. Multi-cellular protease secretion was assumed to represent a uniform protease distribution at 10 nM. In the absence of protease, basal VEGF levels were 1 pM, 5%/40 µm VEGF_165_ gradient (at z = 0) (A). B, C, the steady-state VEGF distribution. The total soluble VEGF (soluble VEGF_165_+VEGF_114_) is exactly identical to the basal state (A). D, E, we considered a range of protease (0–100 nM) and HSPG concentrations (750 nM, 7.5 µM). VEGF_165_ (green), VEGF_114_ (red), total soluble VEGF (blue), and HSPG bound VEGF_165_ conversion (black) (E).

As we have shown that an isolated cell is unable to alter its local soluble VEGF concentration, we attempted to simulate the proteolytic contribution of other cells by simulating VEGF release in a spatially constrained environment. We assume the modeled sprout is one of several sprouts separated by 100 µm (which is mathematically represented by a reflecting boundary at r = 50 µm) simultaneously migrating towards a distant VEGF source (e.g. tumor) (see *Methods*, *Geometrical and Transport Parameters*). We assume that a VEGF gradient is established through a balance of VEGF secretion in front of the sprout and VEGF clearance behind the sprout (see *Methods*, *Implementation*; Table 2) (Fig. 5A). Since we assume protease is secreted simultaneously by numerous cells, we are not concerned with its precise distribution; instead, we are concerned with its mean level and, for simplicity, used a uniform protease concentration field, which does not affect our results (*not shown*).

At a physiological protease level of 10 nM, the conversion of soluble VEGF_165_ is predicted to be approximately 27% (0.97 pM to 0.71 pM), however the total soluble VEGF is unchanged due to a concomitant increase in VEGF_114_ (Fig. 5B, C, E). Because VEGF_165_ is in dynamic equilibrium with HSPG, the total amount of VEGF_165_-HSPG decreases by the same relative amount as VEGF_165_ does (Fig. 5B, D), leading to a decrease in the total VEGF (i.e. bound + soluble) present.

We also find that HSPGs significantly increase VEGF_165_ conversion to VEGF_114_ (Fig. 5D, E), which was also seen in our previous results. To analyze this behavior, we derived effective rates for VEGF proteolysis, internalization, and clearance (refer to *Supplement S1*, *section SVI.2*). We find that HSPGs do not increase the effective rate of VEGF proteolysis, e.g. by providing additional sites for cleavage. Instead HSPGs increase the time VEGF spends in the proteolytic environment, by decreasing the effective VEGF clearance and internalization ∼33-fold in our model. Consequently, our results imply that mechanisms that would increase VEGF clearance or internalization, e.g. increased VEGFR2 expression, would cause VEGF molecules to spend less time in the interstitium and decrease VEGF conversion (*not shown*).

We can similarly use this analysis to estimate the necessary protease concentration for VEGF proteolysis *in vivo*. The useful measure to determine VEGF cleavage in any physiological system is the total clearance rate of a VEGF molecule (i.e. the sum of nonspecific and receptor-mediated mechanisms). Experimental studies indicate a VEGF half-life in the plasma of ∼1 h [42]. Assuming k_P_ = 631 M^−1^s^−1^, ∼305 nM protease would result in 50% VEGF cleavage. These protease levels are however significantly greater than what is expected to be found *in vivo*.

## Discussion

In this study, we developed a computational model of the cellular proteolysis of VEGF. The model includes the secretion of proteases, binding of VEGF to HSPGs and VEGFR2, VEGF_165_ proteolysis to VEGF_114_, and VEGF clearance. While simplified, it allows us to make broad conclusions regarding the extent and determinants of VEGF proteolytic release.

Kinetic fitting of experimental data shows that VEGF cleavage by plasmin is a slow reaction. Using a reaction-diffusion model of a cellular microenvironment, and after overestimating protease secretion (3·10^6^ active protease molecules/cell/h) and accounting for mechanisms of increased cell-surface MMP localization, we find that an isolated cell would have difficulty in causing significant VEGF conversion. Reconciling the *in vivo* observation of VEGF cleavage requires a mechanism of protease accumulation, e.g. geometric constraints and/or simultaneous protease secretion by numerous cells. We thus propose VEGF cleavage is not mediated by single cells but rather by the collective behavior of a tissue, dependent upon total cell density and the whole-tissue MMP concentration.

Our results show that the release of VEGF, mediated by the proteolytic cleavage of matrix-bound VEGF_165_ to soluble VEGF_114_, does not increase total soluble VEGF levels at steady state as long as the total clearance (clearance and internalization) of VEGF_165_ and VEGF_114_ are indistinguishable, which was assumed in our model and was approximately observed in the plasma of mice [42]. In contrast to soluble VEGF, HSPG-bound VEGF levels are lowered in the presence of proteases, reflecting the reduced levels of soluble VEGF_165_ in equilibrium with HSPGs. This effect was especially notable close to the cell surface, for VEGF_165_-HSPG within the range of cell-surface proteases. Thus, we suspect that matrix-bound VEGF may represent a more responsive signaling modality than free VEGF, at least to the presence of proteases. This coupled with a recent finding that matrix-bound VEGF induced prolonged receptor activation compared to soluble VEGF [16] suggests that proteases may be altering angiogenesis primarily through their effects on matrix-bound VEGF. Another interesting possibility to consider is the direct proteolysis of receptor-bound VEGF by cell-surface proteases; due to receptors' slower VEGF dissociation rates (k_off_ = 10^−3^ s^−1^) compared to HSPGs (k_off_ = 10^−2^ s^−1^), they may mediate an even more sensitive protease response curve than that seen with HSPGs (Fig. 4B).

HSPGs are thought to reduce the effective rate of VEGF diffusion by rapidly binding VEGF (theoretically-estimated characteristic time of 3.1 s yielding a 32.5-fold lower effective diffusivity, *Supplement S1 Section SVI.2*). Our model predicts that HSPGs increase the proteolytic conversion of matrix-binding VEGF isoforms by increasing the residence time of a VEGF molecule in a tissue's proteolytic environment. As a result, assuming each VEGF isoform has similar susceptibility to proteases, cleavage of non-HS binding VEGF isoforms (e.g. VEGF_121_, VEGF_165b_) would be expected to be negligible, while cleavage of VEGF_145_ [90] and VEGF_189_ [4], [23] could be even greater than VEGF_165_. In contrast, factors that increase total VEGF clearance, e.g. VEGFR-mediated internalization, result in decreased proteolytic conversion.

Our model makes several important assumptions that are worth clarifying, the most important being the consideration of steady state. Steady state is justified in that the net rate of VEGF clearance *in vivo* is much faster (and hence VEGF reaches a dynamic equilibrium) than structural changes such as vessel reorganization and angiogenesis, which may take several hours to days and are thus relatively static. While our system's overall time scale was ∼10 h (refer to *Supplement S1*, *section SVI.2*), we did not represent the full extent of the vasculature and vessel-mediated internalization in our model. Our previous computational model, representing the whole body microenvironment, estimated a VEGF tissue clearance rate of ∼30 min, primarily due to receptor-mediated internalization [62], consistent with VEGF kinetics in plasma with τ_1/2_∼1 h [42]. Steady state can also be used to determine the time-averaged behavior of tissues, as individual cellular events (e.g. protease secretion by an infiltrated neutrophil and subsequent VEGF release) may be stochastic and transient in nature. This view, in turn, is justified by our results, which show that proteolysis is most likely due to the collective independent behavior of a group of cells.

Another important assumption we made was that HSPGs do not protect VEGF against proteolysis, as seen by Lee et al. for MMP3 [3]. However, other proteases-substrate reactions, e.g. MMP9 and VEGF_164_ [3], or plasmin and bFGF [91], might be sterically blocked by heparan sulfates or HSPGs. In our model, VEGF protection by HSPGs would significantly decrease the VEGF conversion, further raising the necessary *in vivo* protease concentration required to explain the significance of VEGF proteolysis. The role of such proteases may instead be the cleavage of HSPG core protein to enable diffusion of HS-bound VEGF, which was shown for MMP9 in a HT29 colon carcinoma spheroid model [8]. A future analysis of the mechanisms of different proteases should also take into account protease binding to the ECM, which could result in significantly higher tissue protease levels not reflected in serum or plasma levels.

Finally, an endothelial tip cell is structurally more complex more than the simple cylindrical tube we have assumed [13]. Tip cells actively project lamellipodia and filopodia, which increase the surface area of a tip cell, increasing the contact with VEGF in the ECM and possibly facilitating cleavage. Cavities between these extensions may also serve as protected pockets where VEGF and protease activity can be even further localized. In addition, tip cells are more directly exposed to the ECM due to a lack of an intact basement membrane. Our results however suggest that none of these mechanisms significantly enhance VEGF cleavage. An increased tip cell surface area and a loss of a basement membrane facilitate VEGF diffusion away from the cell surface, decreasing the amount of cleaved VEGF present (refer to Fig. 3; *Supplement S1 section SVI.1*). We simulated concentration of proteases to microdomains and the presence of concave pockets (*not shown*); local proteases concentrations do increase, however, not enough to increase pericellular VEGF cleavage to significant levels (*not shown*).

The conventional view of VEGF release is that it is a cell-directed, localized process. VEGF_165_ and VEGF_189_ are typically thought to be tightly bound to HSPGs (e.g. forming deposits of VEGF [3], [13], [53]) that are then rapidly released by pericellular proteolysis, forming an effective autocrine loop, allowing efficient receptor activation of the same cell [19], [53]. Overall, our results show that the rate of VEGF diffusion is exceedingly high to support these views:

### VEGF deposits and the cell-directedness of proteolysis

While the affinity of VEGF_165_ to HSPG is high (24 nM in our study), the dissociation rate is rapid (k_off_ = 0.01 s^−1^) and VEGF_165_ is always near dynamic equilibrium with HSPGs (τ = 3.1 s). This indicates that VEGF_165_ should not form any stationary deposits as they will quickly equilibrate. Deposits of VEGF could instead reflect spatial inhomogeneities in the underlying HSPG itself, which would have a longer lifetime due to the decreased motility of HSPG within the ECM. These inhomogeneities may be important to cellular guidance [53]. In addition, since VEGF proteolysis is slow, VEGF_165_ is more likely to simply dissociate than be cleaved and released in a directed manner by a cell. Directed proteolysis could occur for covalently tethered VEGF [28] and possibly for VEGF_189_ [92], which has high affinity for ECM.

### Localization and extent of pericellular proteolysis

Pericellular proteolysis is significant for many processes [3], [49], [51] and we have previously confirmed this for MT1-MMP mediating collagen cleavage for a migrating cell [50]. However, we argue that this is not the case for catalyzing VEGF_165_ release (or more generally for any ligand of similar diffusivity) (Figs. 2, 3, 4). This is due to VEGF having a much faster rate of replenishment due to diffusion than can be expected for collagen by cell migration [50] (i.e. *Da*_VEGF-Proteolysis_≪*Da*_Col1-Proteolysis_∼1). Moreover, pericellular VEGF cleavage remains insensitive to changes in MMP localization brought about by changes in basement membrane diffusion (Fig. 3) and microdomain sequestration of protease secretion (*not shown*). Due to physiological limits in the number of proteases a cell can secrete and the rate of VEGF cleavage, proteases cannot be both localized to an isolated cell and induce significant VEGF cleavage at the same time. Instead, Fig. 5 shows that for cleaved VEGF to be significant around a cell of interest, the majority of VEGF proteolysis leading to that cleaved VEGF must be occurring outside of the cell's vicinity. This is not to say that proteolysis occurs only in the surrounding ECM; it can also be occurring on the surface of other cells, which was shown by Lee et al. using a coumarin-conjugated VEGF peptide [3]. This also implies that cleaved VEGF must behave in a dispersive fashion, which is discussed in the next section.

### Efficiency of autocrine capture

Even if VEGF is cleaved and released at the cell surface, diffusion away from the cell surface is significantly faster than VEGFR2 binding and internalization (*Supplement S1*, *section SVI.1*): at steady state, the capture probability was only 0.8% for our tip cell with 10^4^ VEGFR2 (*Supplement S1*, *section SVI.1*; *Fig. S3B*). In contrast to our results, a previous analysis [55] demonstrated much higher efficiencies (10–65%) for autocrine capture; however, these probabilities would only arise if the interstitial space were as structurally dense as basement membranes and VEGF diffusivities were as low as 0.1–1 µm^2^/s [54]. Even if we assume that the released VEGF was not cleaved and it can still bind to the ECM, we show that the diffusive hindrance due to HSPGs does not increase autocrine capture (only diffusive hindrances due to matrix tortuosity and water/fiber hydrodynamic interactions [93] yield increases in autocrine capture). By sequestering VEGF away from receptors, HSPGs decrease the transient capture of VEGF by VEGFR2 (*not shown*) and have no effect at steady state. Finally, our results imply that autocrine VEGF release and recapture would be even less likely in the presence of interstitial flow. This contradicts the hypothesis of a previous finding that the autocrine detection of convection-driven gradients of proteolytically-released VEGF may enhance capillary morphogenesis [19].

**Figure 6:**
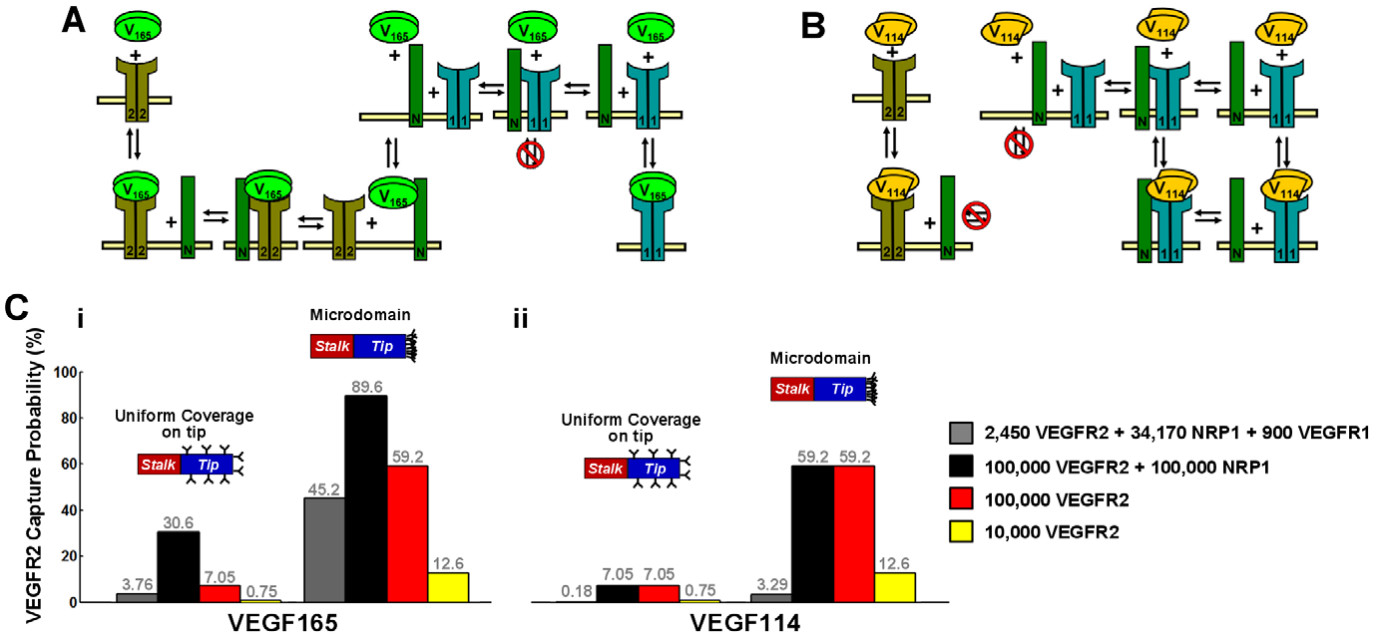
VEGF receptors on autocrine capture by a tip cell. We estimated autocrine capture probabilities for a VEGF molecule placed at the cell surface. Overall capture probabilities through all receptors were estimated using a continuum approximation, P_cap_ = 1−[VEGF]_r = Rcell_/[VEGF]_r = Inf_. We assumed an isolated cell (case 1, with no receptors on the stalk cells), set far-field [VEGF] = 1 pM, and measured cell surface [VEGF] after the steady state in VEGF internalization. Capture probability by VEGFR2 was further approximated by multiplying P_cap_ by the ratio of receptor-bound VEGF bound to VEGFR2. A, B, schematic of VEGF_165_ and VEGF_114_ interactions with VEGFR1, VEGFR2, and NRP1. Equations for the full reaction network are given in *Supplement S1*, *section SII*. C, capture probability of VEGF_165_ (i) and VEGF_114_ (ii) by VEGFR2. We considered two tip cell receptor distributions: uniform coverage and localization to the front edge (microdomain). Gray bars indicate receptor numbers recently measured on HUVECs (P. Imoukhuede, *personal communication*); other colors represent reference cases.

Autocrine capture can be increased by several mechanisms, including increasing the thickness of a surrounding basement membrane (Fig. 3). For a tip cell, which has at best a tenuous basement membrane, capture is more likely to be increased by an increase in receptor expression [13] and by spatially restricting receptor and protease activity to discrete microdomains on the cell surface (Fig. 6). For example, a uniform distribution of 10^5^ NRP1 and 10^5^ VEGFR2 can extend the a tip cell's capture of VEGF_165_ to ∼30%, while clustering all receptors to the leading edge of the tip cell further increases capture to ∼87%. Capture of cleaved VEGF is weaker than that of VEGF_165_ due to a lack of NRP1-binding, and for typical receptor concentrations found in HUVECs (Fig. 6C, gray bars), remains negligible. This implies that VEGF isoforms released through ECM cleavage (thus still maintaining their NRP1- and cell-surface HS-binding domains) will experience higher autocrine capture probabilities than VEGF released through VEGF cleavage.

**Figure 7:**
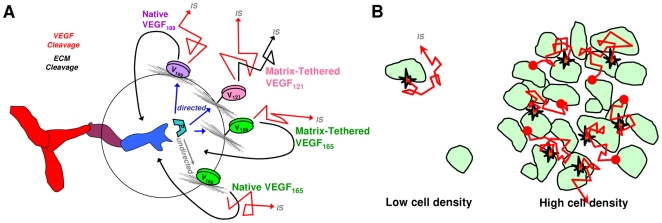
VEGF proteolysis and cell capture. A cell's ability to direct and utilize VEGF release depends on both the ability to release VEGF and recapture VEGF. A, we compare different forms of VEGF, all equidistant from a hypothetical tip cell. The directedness of release depends on the strength of VEGF binding to the ECM. VEGF_165_ rapidly dissociates and cannot be directed, while release of VEGF_189_ and matrix-tethered formulations may be directed. Autocrine capture requires that the released VEGF bind to receptors efficiently (potentiated by NRP1), thus cleaved VEGF or VEGF_121_ will not be suitable for autocrine signaling. Instead, cleaved VEGF will diffuse away into the interstitial space (IS), operating in a paracrine fashion, B. A high cell density ensures that VEGF is efficiently cleaved (due to accumulation of proteases) and recaptured within a tissue.

Overall, proteolysis can be cell-directed only for strongly-matrix-binding isoforms, while further autocrine activity can only occur if VEGF is liberated in a fashion that maintains its co-receptor-binding domains (Fig. 7A). The extent of VEGF cleavage is significant only at higher cell densities and cleaved VEGF necessarily acts in a dispersive, paracrine manner (Fig. 7B).

Since VEGF cleavage is a multi-cellular phenomenon, its effectiveness for any single cell capable of sensing VEGF depends on the total density of cells secreting or cleaving VEGF, as well as overall tissue clearance and receptor-mediated uptake rates. While our model did not explicitly account for the multiple cell types involved in angiogenesis (e.g. macrophages, pericytes, and parenchymal cells) [32], [94], our results further support the concept that numerous cells and cell types are involved in the angiogenic response. In cell cultures, a low cell density environment, we expect a low fraction of existing VEGF to be released (i.e. low conversion), shown experimentally in [14], though in at least one case, significant levels of cleaved VEGF were observed in the conditioned media of ovarian cancer cell lines [33]. At high cell densities, such as *in vivo* (e.g. tumors, oxygen-induced retinopathy), VEGF conversion could be significant (resulting in a significant fraction of plasma VEGF being cleaved) [3], [17]. However, our preliminary estimates require ∼305 nM active protease to account for this high level of cleaved VEGF, concentrations that are greatly in excess of physiological or pathological protease levels. To resolve this paradox, it must be determined whether all known proteases are taken into account (e.g. matrix-sequestered MMPs), as well as to determine the accuracy of our estimated kinetic parameters of VEGF cleavage.

### Conclusion

We developed a computational model to simulate the proteolytic cleavage of VEGF in the vicinity of an endothelial sprout. We found that VEGF proteolysis by plasmin is slow compared to other proteolysis reactions. In order for VEGF conversion to be significant, we required a model where protease production is not limited to a single cell. Our results suggest autocrine effects can only be supported by ECM-cleaving VEGF release, which would preserve VEGF's ability to bind to co-receptors, while paracrine effects are expected for VEGF-cleaving VEGF release. Localization of proteases and receptors to cell surface microdomains can significantly improve autocrine capture, however it has no significant effect on VEGF proteolysis. Our analysis also shows two distinct roles for HSPGs: they increase VEGF conversion by decreasing the overall VEGF clearance rate in tissues and also provide a unidirectional signaling modality in the presence of proteases. Future experiments should test the cell density dependence on VEGF cleavage as well more accurately estimate the quantity of proteases present *in vivo*.

## Supporting Information

Supplement S1 (0.60 MB DOC)
