# Supplementary material for: Quantifying the Proteolytic Release of Extracellular Matrix-Sequestered VEGF with a Computational Model
Source: PLoS One. 2010 Jul 29;5(7):e11860. doi: 10.1371/journal.pone.0011860 (PMC2912330; doi:10.1371/journal.pone.0011860)
Supplement: Supplement S1 — (0.60 MB DOC) [file pone.0011860.s001.doc]

**Supplemental Methods, Results, and Discussion**

The supplemental material is used to provide in-depth methods, derivation, or calculation of auxiliary results. They are organized by topic, as below:

Section I. Determination of kinetic parameters for VEGF cleavage by plasmin

Section II. Full equations for VEGF isoform interactions with VEGFR2, VEGFR1, and NRP1

Section III. Differences between a two-step proteolysis and one-step proteolysis

Section IV. Numerical Methods for effective mass transfer through the basement membrane layer

Section V. Estimation of VEGF diffusion hindrance in the ECM and BM

Section VI. Analytical expressions for VEGF transport and cell-surface proteolysis

**Section SI. Determination of kinetic parameters for VEGF cleavage by plasmin**

VEGF cleavage by plasmin was monitored using repetitive reverse-phase high performance liquid chromatography (HPLC) at 25°C [1], where the VEGF_165_ homodimer was found to undergo sequential cleavage from VEGF_165-165_, to VEGF_165-110_, and finally to VEGF_110-110_:

VEGF_165-165_ + Plasmin → VEGF_165-110_ + Plasmin

VEGF_165-110_ + Plasmin → VEGF_110-110_ + Plasmin

The experimental data is given in Fig. S1A. The initial VEGF_165-165_ and plasmin concentrations were 670 μM (30 μg in 10 μL aliquots) and 350 nM (0.3 μg in 10 μL), respectively, and the proteolysis was carried out for 12 h [1]. We numerically fit the data to derive kinetic rate parameters using an iterative grid search. The model was described as ordinary differential equations using mass-action kinetics, assuming either an effective single-step or Michaelis-Menten scheme for each step of the conversion. For example, each step of the sequential reaction can be described using a single-step scheme:

$$\frac{\text{d[V}_{165-165}\text{]}}{\text{dt}}=-\text{k}_{\text{P}}^{\text{165-165}}\text{[P][V}_{165-165}\text{]} \text{(SI-1)}$$

$$\frac{\text{d[V}_{165-110}\text{]}}{\text{dt}}=\text{k}_{\text{P}}^{\text{165-165}}\text{[P][V}_{165-165}\text{]}-\text{k}_{\text{P}}^{\text{165-110}}\text{[P][V}_{165-110}\text{]} \text{(SI-2)}$$

$$\frac{\text{d[V}_{110-110}\text{]}}{\text{dt}}=\text{k}_{\text{P}}^{\text{165-110}}\text{[P][V}_{165-110}\text{]} \text{(SI-3)}$$

Assuming that VEGF_165-165_ is cleaved at twice the rate of VEGF_165-110_, we fit the data to the above sequential reaction model yielding bimolecular rate constants k_P_^165-165^ = 656 M^−1^s^−1^ for VEGF_165-165_ and k_P_^165-110^ = 328 M^−1^s^−1^ for VEGF_165-110_ (Fig. S1A). Compared to the single-step scheme, a Michaelis-Menten scheme did not achieve a significantly better fit (*data not shown*), implying that [VEGF] = 670 μM likely represents a nonsaturating substrate concentration, i.e. K_m_ >> 670 μM. Keyt et al. reported a relative rate of 3:1 for proteolysis of the homodimer to heterodimer [1]. We found that this ratio could also fit the experimental data, albeit for different intrinsic rate constants. However, our analysis shows that the 2:1 ratio is also sufficient to explain the observed kinetics.

Since the heterodimer was shown to have properties intermediate to the VEGF_165-165_ and VEGF_110-110_ homodimers in receptor binding, heparin-sepharose elution, and cell mitogenicity assays [1], we reduced the two sequential reactions into an effective one-step reaction from VEGF_165-165_ to VEGF_110-110_ (see *SIII* for comparison of the two-step and one-step reactions). The effective single-step rate constant then becomes k_P_ = 328 M^−1^s^−1^ (Fig. S1B). We further scaled this to 37°C using the Arrhenius equation, assuming an activation energy of E_A_ ~ 10^4^ cal/mol (typical of many enzymatic reactions [2]), to obtain k_P_ = 631 M^−1^s^−1^. This kinetic parameter is representative of the low end of typical ECM enzyme-substrate reactions (Table *S1*). Furthermore, since plasmin and MMP3 seem to cleave VEGF_164_ at similar molar rates (see supplement of [3]), we assume that k_P_ = 631 M^−1^s^−1^ also describes VEGF_165_ cleavage to VEGF_114_ by MMP3.

We also briefly examined the kinetics of VEGF cleavage for typical *in vivo* and *in vitro* MMP concentrations at the low nM range [4,5], assuming a well-stirred reactor vessel (Fig. S1C). For example, 10 nM MMP3 yields a time-constant of τ = (k_P_∙[Protease])^−1^ = 44 h.


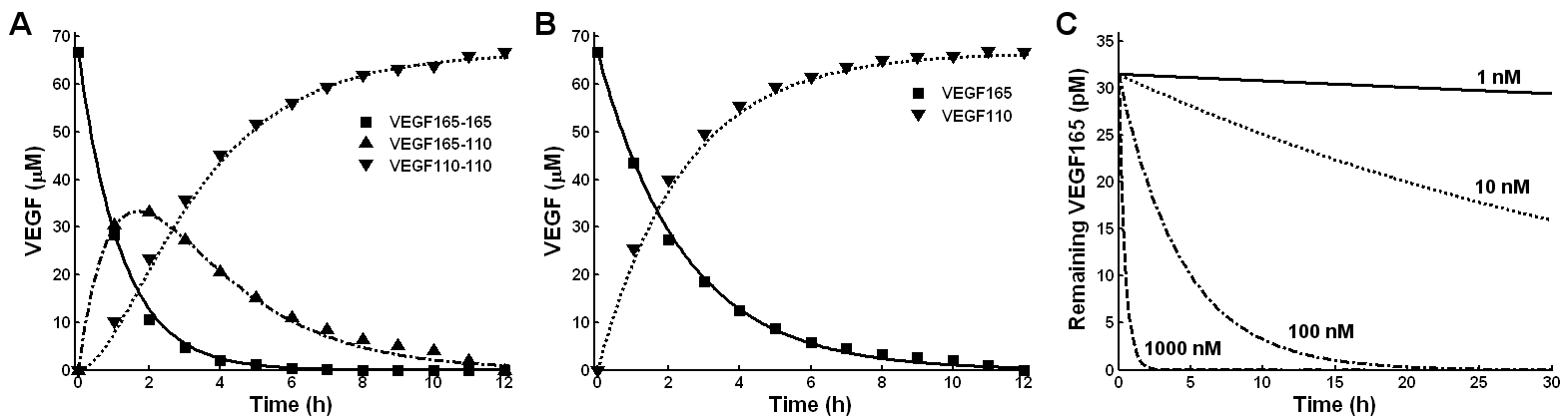


Figure S1 – Characterization of VEGF cleavage by plasmin. We estimated the kinetic parameters for VEGF cleavage by plasmin using experimental data from Keyt et al. [1] (markers in A). In their experiment, 30 μg VEGF_165-165_ homodimer was reacted with 0.3 μg plasmin in a 10 μL aliquot; the time course of cleavage was measured using reverse phase high performance liquid chromatography (HPLC) at 25°C. Lines: theoretical simulations with k_P_^165-165^ ~ 656 M^−1^s^−1^, k_P_^165-110^ ~ 328 M^−1^s^−1^. B, overall proteolytic reaction simplified into a one-step reaction (VEGF_165_ 🡪 VEGF_110_) with k_P_ = 328 M^−1^s^−1^. Total [VEGF_165_] was measured as [VEGF_165-165_] + 0.5∙[VEGF_165-110_] and [VEGF_110_] as [VEGF_110-110_] + 0.5∙[VEGF_165-110_]; the experimental data was also similarly calculated. C, VEGF (maintained with a soluble concentration of 1 pM and equilibrated with 750 nM HSPG) is cleaved by an effective one-step reaction. The kinetics were approximated to 37°C by scaling using the Arrhenius relation and an E_A_ ~ 10^4^ cal/mol (resulting in k_P_ = 631 M^−1^s^−1^) [2].

Table S1. Kinetic Parameters of ECM Cleavage

| **Substrate** | **Protease** | **K_m_** | **k_cat_** | **k_cat_/K_m_** | **T (°C)** | **Ref.** |
| --- | --- | --- | --- | --- | --- | --- |
| Decorin | MMP2 | K_m_ = 10 μM | k_cat_ = 0.018 s^−1^ | 1.8∙10^3^ M^−1^s^−1^ | 37 | [6] |
| Decorin | MMP3 | K_m_ = 12 μM | k_cat_ = 0.022 s^−1^ | 1.8∙10^3^ M^−1^s^−1^ | 37 | [6] |
| Entactin | MMP7 | K_m_ = 0.89 μM | k_cat_ = 0.35 s^−1^ | 390∙10^3^ M^−1^s^−1^ (E_A_ = 10,060 cal/mol) | 37 | [2] |
| Type I Collagen | MMP1 | K_m_ = 0.45 μM | k_cat_ = 0.0096 s^−1^ | 21∙10^3^ M^−1^s^−1^ | 37 | [7]† |
| Type I Collagen | MMP1 | K_m_ = 0.90 μM | k_cat_ = 0.0054 s^−1^ | 6.0∙10^3^ M^−1^s^−1^ (E_A_ = 101,050 cal/mol) | 25 | [8] |
| Type I Collagen | MMP2 | K_m_ = 8.5 μM | k_cat_ = 0.0045 s^−1^ | 5.3∙10^3^ M^−1^s^−1^ | 25 | [9] |
| Type I gelatin (α chain) | MMP2 | K_m_ = 7 μM | k_cat_ = 6.5 s^−1^ | 900∙10^3^ M^−1^s^−1^ | 37 | [10] |
| Type III Collagen | MMP1 | K_m_ = 1.3 μM | k_cat_ = 0.15 s^−1^ | 120∙10^3^ M^−1^s^−1^ | 25 | [11] |
| Type V Collagen | MMP9 | K_m_ ~ 5 μM | k_cat_ = 0.011 s^−1^ | 2∙10^3^ M^−1^s^−1^ | 32 | [12] |

Legend:

† Theoretical estimate

**Section SII. Full equations for VEGF isoform interactions with VEGFR2, VEGFR1, and NRP1**

The interactions of VEGF_165_ and VEGF_114_ with VEGFR2, VEGFR1, and NRP1 are portrayed in Fig. 6A, B of the manuscript. These interactions take place in the basement membrane layer of our model and are represented as ordinary differential equations. Along with the equations describing VEGF_165_-HSPG, HSPG, and Protease, the full set of equations governing the basement membrane layer species are:

*Interstitial Species*

$$\begin{matrix} \frac{d\text{[V}_{165}\text{]}}{dt}=-\frac{\text{J}_{\text{out}}^{\text{V165}}}{\text{d}_{\text{BM}}}-\frac{\text{k}_{\text{on}}}{\text{K}_{\text{BM}}}\text{[V}_{165}\text{][H]}+\text{k}_{\text{off}}\text{[V}_{165}\text{H]}-\frac{\text{k}_{\text{P}}}{\text{K}_{\text{BM}}}\text{[V}_{165}\text{][P]}-\frac{\text{k}_{\text{on}}^{\text{165, R2}}}{K_{BM}}\text{[V}_{165}\text{]}\frac{\text{[R}2]}{\text{d}_{\text{BM}}}+\text{k}_{\text{off}}^{\text{165, R2}}\frac{\text{[V}_{165}\text{R2]}}{\text{d}_{\text{BM}}} \\ \text{ }-\frac{\text{k}_{\text{on}}^{\text{165, R1}}}{K_{BM}}\text{[V}_{165}\text{]}\frac{\text{[R}1]}{\text{d}_{\text{BM}}}+\text{k}_{\text{off}}^{\text{165, R1}}\frac{\text{[V}_{165}\text{R1]}}{\text{d}_{\text{BM}}}-\frac{\text{k}_{\text{on}}^{\text{165, N1}}}{K_{BM}}\text{[V}_{165}\text{]}\frac{\text{[N}1]}{\text{d}_{\text{BM}}}+\text{k}_{\text{off}}^{\text{165, N1}}\frac{\text{[V}_{165}\text{N1]}}{\text{d}_{\text{BM}}} \text{(SII-1)} \end{matrix}$$

$$\begin{matrix} \frac{d\text{[V}_{114}\text{]}}{dt}=-\frac{\text{J}_{\text{out}}^{\text{V114}}}{\text{d}_{\text{BM}}}+\frac{\text{k}_{\text{P}}}{\text{K}_{\text{BM}}}\text{[P][V}_{165}\text{]} +\frac{\text{k}_{\text{P}}}{\text{K}_{\text{BM}}}\text{[P][V}_{165}\text{H]} -\frac{\text{k}_{\text{on}}^{\text{114, R2}}}{K_{BM}}\text{[V}_{114}\text{]}\frac{\text{[R}2]}{\text{d}_{\text{BM}}}+\text{k}_{\text{off}}^{\text{114, R2}}\frac{\text{[V}_{114}\text{R2]}}{\text{d}_{\text{BM}}} \\ -\frac{\text{k}_{\text{on}}^{\text{114, R1}}}{K_{BM}}\text{[V}_{114}\text{]}\frac{\text{[R}1]}{\text{d}_{\text{BM}}}+\text{k}_{\text{off}}^{\text{114, R1}}\frac{\text{[V}_{114}\text{R1]}}{\text{d}_{\text{BM}}}-\frac{\text{k}_{\text{on}}^{\text{114, R1N1}}}{K_{BM}}\text{[V}_{114}\text{]}\frac{\text{[R1N}1]}{\text{d}_{\text{BM}}}+\text{k}_{\text{off}}^{\text{114, R1N1}}\frac{\text{[V}_{114}\text{R1N1]}}{\text{d}_{\text{BM}}} \text{(SII-2)} \end{matrix}$$

$$\frac{\text{d[V}_{165}\text{H]}}{\text{dt}}=\frac{\text{k}_{\text{on}}}{\text{K}_{\text{BM}}}\text{[V}_{165}\text{][H]}-\text{k}_{\text{off}}\text{[V}_{165}\text{H]}-\frac{\text{k}_{\text{P}}}{\text{K}_{\text{BM}}}\text{[V}_{165}\text{H][P]} (SII-4)$$

$$\frac{\text{d[H]}}{\text{dt}}=-\frac{\text{k}_{\text{on}}}{\text{K}_{\text{BM}}}\text{[V}_{165}\text{][H]}+\text{k}_{\text{off}}\text{[V}_{165}\text{H]}+\frac{\text{k}_{\text{P}}}{\text{K}_{\text{BM}}}\text{[V}_{165}\text{H][P]} (SII-3)$$

$$\frac{\text{d[P]}}{\text{dt}}= \frac{\text{q}_{\text{P}}-\text{J}_{\text{out}}^{\text{P}}-k_{\deg}[P]}{\text{d}_{\text{BM}}} (SII-5)$$

*Membrane-Bound Species*

$$\begin{matrix} \frac{d\text{[R1]}}{dt}=\text{s}_{\text{R1}}-\text{k}_{\text{int}}^{\text{R1}}\text{[R1]}-\frac{\text{k}_{\text{on}}^{\text{165,R1}}}{K_{BM}}\text{[V}_{165}\text{][R1]}+\text{k}_{\text{off}}^{\text{165,R1}}\text{[V}_{165}\text{R1]}-\frac{\text{k}_{\text{on}}^{\text{114,R1}}}{K_{BM}}\text{[V}_{114}\text{][R1]}+\text{k}_{\text{off}}^{\text{114,R1}}\text{[V}_{114}\text{R1]} \\ -\text{k}_{\text{c}}^{\text{R1,N1}}\text{[R1][N1]}+\text{k}_{\text{uc}}^{\text{R1,N1}}\text{[R1N1]} \text{(SII-6)} \end{matrix}$$

$$\begin{matrix} \frac{d\text{[R2]}}{dt}=\text{s}_{\text{R2}}-\text{k}_{\text{int}}^{\text{R2}}\text{[R2]}-\frac{\text{k}_{\text{on}}^{\text{165,R2}}}{K_{BM}}\text{[V}_{165}\text{][R2]}+\text{k}_{\text{off}}^{\text{165,R2}}\text{[V}_{165}\text{R2]}-\frac{\text{k}_{\text{on}}^{\text{114,R2}}}{K_{BM}}\text{[V}_{114}\text{][R2]}+\text{k}_{\text{off}}^{\text{114,R2}}\text{[V}_{114}\text{R2]} \\ -\text{k}_{\text{c}}^{\text{165N1,R2}}\text{[V}_{165}\text{N1][R2]}+\text{k}_{\text{uc}}^{\text{165N1,R2}}\text{[V}_{165}\text{R2N1]} \text{(SII-7)} \end{matrix}$$

$$\begin{matrix} \frac{d\text{[N1]}}{dt}=\text{s}_{\text{N1}}-\text{k}_{\text{int}}^{\text{N1}}\text{[N1]}-\frac{\text{k}_{\text{on}}^{\text{165,N1}}}{K_{BM}}\text{[V}_{165}\text{][N1]}+\text{k}_{\text{off}}^{\text{165,N1}}\text{[V}_{165}\text{N1]}-\text{k}_{\text{c}}^{\text{165R2, N1}}\text{[V}_{165}\text{R2][N1]}+\text{k}_{\text{uc}}^{\text{165R2,N1}}\text{[V}_{165}\text{R2N1]} \\ -\text{k}_{\text{c}}^{\text{114R1, N1}}\text{[V}_{114}\text{R1][N1]}+\text{k}_{\text{uc}}^{\text{114R1,N1}}\text{[V}_{114}\text{R1N1]}-\text{k}_{\text{c}}^{\text{R1,N1}}\text{[R1][N1]}+\text{k}_{\text{uc}}^{\text{R1,N1}}\text{[R1N1]} \text{(SII-8)} \end{matrix}$$

$$\begin{matrix} \frac{d\text{[R1N1]}}{dt}=-\text{k}_{\text{int}}^{\text{VR1N1}}\text{[R1N1]}+\text{k}_{\text{c}}^{\text{R1,N1}}\text{[R1][N}1\text{]}-\text{k}_{\text{uc}}^{\text{VR1,N1}}\text{[R1N1]} \\ -\frac{\text{k}_{\text{on}}^{\text{114, R1N1}}}{K_{BM}}\text{[V}_{114}\text{][R1N}1\text{]}+\text{k}_{\text{off}}^{\text{114, R1N1}}\text{[V}_{114}\text{R1N}1\text{]} \text{(SII-9)} \end{matrix}$$

$$\frac{d\text{[V}_{165}\text{R1]}}{dt}=-\text{k}_{\text{int}}^{\text{165R1}}\text{[V}_{165}\text{R1]}+\frac{\text{k}_{\text{on}}^{\text{165, R1}}}{K_{BM}}\text{[V}_{165}\text{][R1]}-\text{k}_{\text{off}}^{\text{165, R1}}\text{[V}_{165}\text{R1]} \text{(SII-10)}$$

$$\begin{matrix} \frac{d\text{[V}_{165}\text{R2]}}{dt}=-\text{k}_{\text{int}}^{\text{165R2}}\text{[V}_{165}\text{R2]}+\frac{\text{k}_{\text{on}}^{\text{165, R2}}}{K_{BM}}\text{[V}_{165}\text{][R}2\text{]}-\text{k}_{\text{off}}^{\text{165, R2}}\text{[V}_{165}\text{R2]} \\ -\text{k}_{\text{c}}^{\text{165R2,N1}}\text{[V}_{165}\text{R2][N}1\text{]}+\text{k}_{\text{uc}}^{\text{165R2,N1}}\text{[V}_{165}\text{R2N1]} \text{(SII-11)} \end{matrix}$$

$$\begin{matrix} \frac{d\text{[V}_{165}\text{N1]}}{dt}=-\text{k}_{\text{int}}^{\text{165N1}}\text{[V}_{165}\text{N1]}+\frac{\text{k}_{\text{on}}^{\text{165, N1}}}{K_{BM}}\text{[V}_{165}\text{][N1]}-\text{k}_{\text{off}}^{\text{165, N1}}\text{[V}_{165}\text{N1]} \\ -\text{k}_{\text{c}}^{\text{165N1,R2}}\text{[V}_{165}\text{N1][R2]}+\text{k}_{\text{uc}}^{\text{165N1,R2}}\text{[V}_{165}\text{R2N1]} \text{(SII-12)} \end{matrix}$$

$$\begin{matrix} \frac{d\text{[V}_{165}\text{R2N1]}}{dt}=-\text{k}_{\text{int}}^{\text{165R2N1}}\text{[V}_{165}\text{R2N1]}+\text{k}_{\text{c}}^{\text{165R2, N1}}\text{[V}_{165}\text{R2][N}1]-\text{k}_{\text{uc}}^{\text{165R2,N1}}\text{[V}_{165}\text{R2N1]} \\ +\text{k}_{\text{c}}^{\text{165N1,R2}}\text{[V}_{165}\text{N1][R}2]-\text{k}_{\text{uc}}^{\text{165N1,R2}}\text{[V}_{165}\text{R2N1]} \text{(SII-13)} \end{matrix}$$

$$\begin{matrix} \frac{d\text{[V}_{114}\text{R1]}}{dt}=-\text{k}_{\text{int}}^{\text{114R1}}\text{[V}_{114}\text{R1]}+\frac{\text{k}_{\text{on}}^{\text{114, R1}}}{K_{BM}}\text{[V}_{114}\text{][R}1]-\text{k}_{\text{off}}^{\text{114, R1}}\text{[V}_{114}\text{R1]} \\ -\text{k}_{\text{c}}^{\text{114R1,N1}}\text{[V}_{114}\text{R1][N}1]+\text{k}_{\text{uc}}^{\text{114R1,N1}}\text{[V}_{114}\text{R1N1]} \text{(SII-14)} \end{matrix}$$

$$\frac{d\text{[V}_{114}\text{R2]}}{dt}=-\text{k}_{\text{int}}^{\text{114R2}}\text{[V}_{114}\text{R2]}+\frac{\text{k}_{\text{on}}^{\text{114, R2}}}{K_{BM}}\text{[V}_{114}\text{][R}2]-\text{k}_{\text{off}}^{\text{114, R2}}\text{[V}_{114}\text{R2]} \text{(SII-15)}$$

$$\begin{matrix} \frac{d\text{[V}_{114}\text{R1N1]}}{dt}=-\text{k}_{\text{int}}^{\text{114R1N1}}\text{[V}_{114}\text{R1N1]}+\frac{\text{k}_{\text{on}}^{\text{114, R1N1}}}{K_{BM}}\text{[V}_{114}\text{][R1N}1]-\text{k}_{\text{off}}^{\text{114, R1N1}}\text{[V}_{114}\text{R1N}1] \\ +\text{k}_{\text{c}}^{\text{114R1,N1}}\text{[V}_{114}\text{R1][N}1]-\text{k}_{\text{uc}}^{\text{114R1,N1}}\text{[V}_{114}\text{R1N1]} \text{(SII-16)} \end{matrix}$$

The rate constants for the additional VEGFR1- and NRP1-related reactions have been given previously [13]. The rate constants for association-dissociation of VEGF_165_ or VEGF_114_ to VEGFR2 are k_on_ = 10^7^ M^−1^s^−1^, k_off_ = 10^−3^ s^−1^. Similarly, for VEGFR1, we have k_on_ = 3∙10^7^ M^−1^s^−1^, k_off_ = 10^−3^ s^−1^. Only VEGF_165_ interacts with NRP1, at k_on_ = 3.2∙10^6^ M^−1^s^−1^ and k_off_ = 0.001 s^−1^, while only VEGF_114_ can interact with VEGFR1-NRP1, which it does with the same kinetics as it interacts with VEGFR1. The coupling-uncoupling rates of VEGF_165_-VEGFR2 to NRP1 are k_c_ = 3.1∙10^6^ (10^15^ μm^2^)/(mol∙s) and k_uc_ = 10^−3^ s^−1^ while those for VEGF_165_-NRP1 to VEGFR2 are k_c_ = 10^7^ (10^15^ μm^2^)/(mol∙s) and k_uc_ = 10^−3^ s^−1^. The coupling-uncoupling rates of VEGFR1 and VEGF_114_-VEGFR1 to NRP1 are k_c_ = 10^7^ (10^15^ μm^2^)/(mol∙s) and k_uc_ = 10^−2^ s^−1^. Finally, we assume all internalization rate constants, k_int_, are identical at 2.8∙10^−4^ s^−1^, while receptor insertion rates, s_R2_, s_R1_, s_N1_, are set so as to constantly maintain the desired total number of receptors at the cell surface, i.e. s_R2_ = k_int_^R2^∙[R2]_Total_, etc.

**Section SIII. Differences between a two-step proteolysis and one-step proteolysis**

Keyt et al. showed that the VEGF_165-165_ homodimer is converted to a VEGF_110-110_ homodimer via the intermediate VEGF_165-110_ (two-step scheme). In our model, we assumed a one-step proteolysis scheme where VEGF_165-165_ is converted directly into VEGF_114-114_. Our model’s simplified approximation can be justified as testing the two-step scheme shows that the results do not significantly differ (see Fig. S2 below) (the timescale of the process is changed by ~3-fold). We assume the intermediate VEGF_165-114_ has half the affinity as the VEGF_165-165_ homodimer for HSPG and Neuropilin-1 binding. In this case for the two-step proteolysis model, we have to define the effective soluble VEGF_165_ as VEGF_165-165_ + ½*VEGF_165-114_ and the effective soluble VEGF_114_ as VEGF_114_ + ½*VEGF_165-114_. The total effective bound VEGF is the equal to bound VEGF_165-165_ + bound VEGF_165-114_. One difference in the two-step model that is not found in the one-step model is that VEGF_114_ activity can be bound to HSPGs (via VEGF_114-165_) and presumably Neuropilin-1. This would then present differences in situations where VEGFRs and Neuropilins can ligate VEGF from its ECM- or BM-bound state. Another justification for our one-step simplification is that we simplify most other reactions to one-step and single-component processes, e.g. VEGFR2 binding, HSPG binding, etc. In reality, for example, HSPGs would represent a heterogeneous affinity binding population for VEGF for which the specific fractions and binding kinetics are not known.


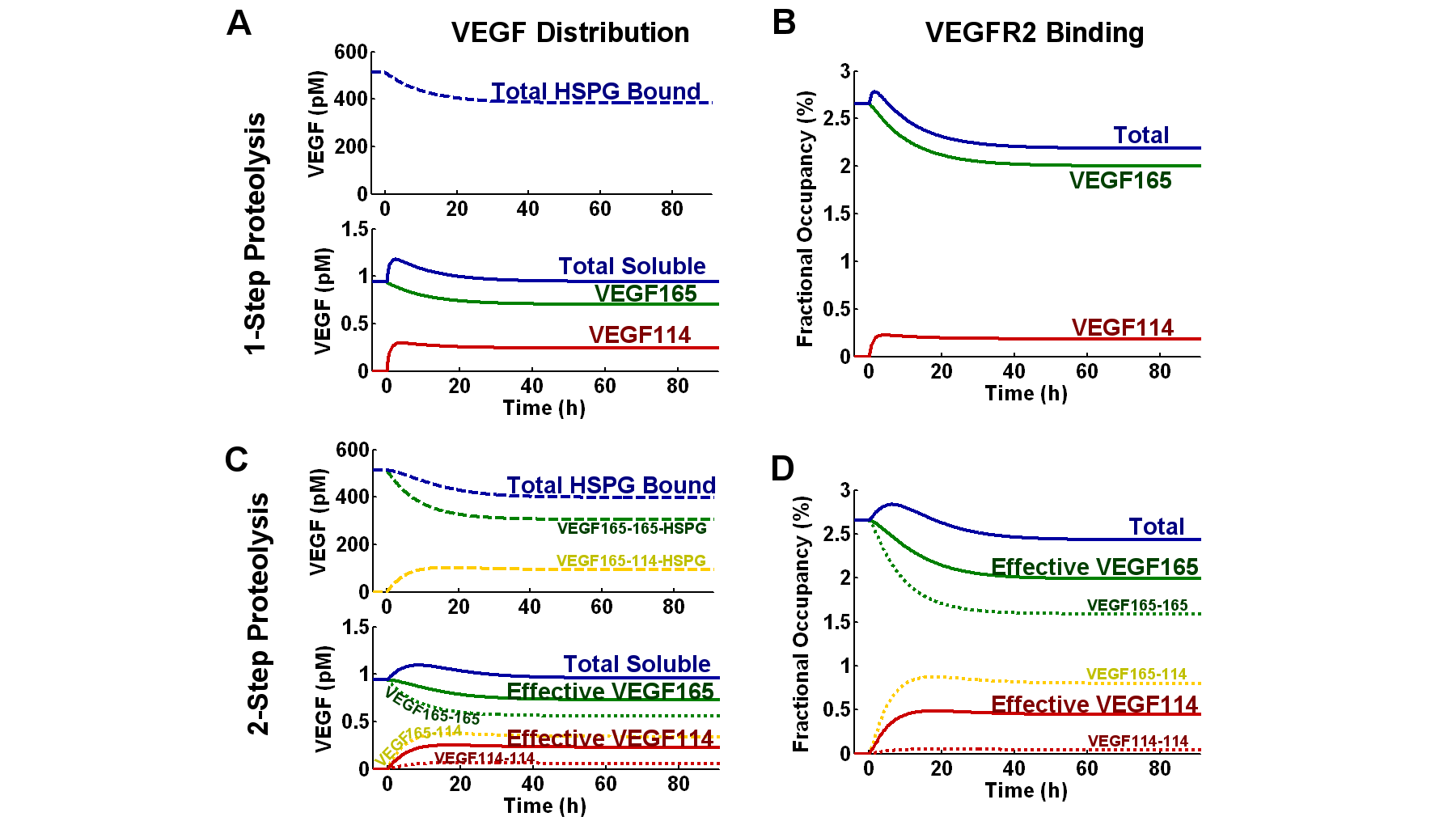


Figure S2 – Comparison of a two-step versus a one-step proteolysis scheme. The time-course of the pericellular VEGF concentration (A, C) and VEGFR2 fractional occupancy (B, D) is shown for a two-step and one-step scheme. We assumed a case-2, non-isolated, cell and an extracellular protease concentration of 10 nM uniformly imposed in the domain at t = 0 and held constant. We specified 10^4^ VEGFR1, 10^4^ VEGFR2, and 10^4^ Neuropilin-1 on the tip cell. Neuropilin-1 was specified because it differs in its binding between VEGF_165_, VEGF_114_, and the heterodimer and presents a stringent comparison of the two models. In the two-step model, we calculated the effective concentrations and receptor binding of soluble VEGF_165_ (VEGF_165-165_ + ½*VEGF_165-114_) and VEGF_114_ (VEGF_114-114_ + ½*VEGF_165-114_) for direct comparison against the one-step model. Similarly, total bound VEGF in the two-step model was given by the sum of VEGF_165-165_-HSPG and VEGF_165-114_-HSPG. We find that the steady-state distributions of the two-step and one-step mechanisms are similar, while the overall kinetics of the two-step reaction is slower.

**Section SIV. Numerical methods for effective mass transfer through the basement membrane layer**

The basement membrane layer is extremely thin (~43 nm) and the dynamics in this layer is treated as a lumped boundary condition for the solution of the PDEs within the ECM region. The derivation of this formulation begins with the integration of the PDEs describing this system over the thickness of the basement membrane layer (from the cell surface to the interface of the basement membrane and ECM):

Here, R_C_ represents the net rate of reaction of C, d_BM_ is the thickness of the basement membrane layer, $\bar{C}$is the spatial average of the concentration of C, and K_BM_ is the available volume fraction of the basement membrane layer. The term $R_{\bar{C}}$ represents the spatial average of the reaction rate, which we approximate as the rate of reaction at the average concentration$\bar{C}$. We assume that the cell-surface receptors can probe the entirety of the basement membrane layer so that in essence, they sense the concentration $\bar{C}$. The diffusive flux at the cell surface is given by the secretion rate of C while the diffusive flux from the basement membrane to the ECM is defined as J:

As q and $R_{\bar{C}}$ are known, our goal is to provide a numerical approximation for J. n = d_BM-_ represents the interface of the basement membrane layer and ECM as approached from the cell surface. To evaluate $\left.J\right|_{d_{BM^{-}}}$, we impose flux continuity between the ECM and basement membrane layer. We create a hypothetical point at the interface with bulk concentration $\left.C\right|_{d_{BM}}$ and specify x_1_ as the first grid node in the ECM away from the basement membrane surface. Thus, the flux continuity becomes

In the above equations, flux is written in a general form that accounts for the possibility of spatial variation in K_BM_ and K_ECM_. Since we also have a continuity in interstitial fluid concentrations, i.e. $\left.C\right|_{d_{BM}^{-}}=\left.K_{BM}/K_{ECM}\cdot C\right|_{d_{BM}^{+}}$, we get that

Where α = D_C_^BM^/(d_BM_/2), β = D_C_^ECM^/(x_1_ – d_BM_), and γ = K_ECM_/K_BM_.

As a result, the flux of C from the basement membrane to the ECM (i.e. J_out_ in Eqn. 8 of the manuscript) is given by

$$\left.J\right|_{d_{BM}}=-\frac{\alpha\beta\gamma}{\alpha+\beta\cdot\gamma}\left(\left.C\right|_{x_{1}}\frac{1}{\gamma}-\bar{C}\right)() (SIV-4)$$

**Section SV. Estimation of VEGF diffusion hindrance in the ECM and the basement membrane layer**

To calculate the diffusivity of molecules in the ECM and basement membrane matrices, we followed a procedure outlined in previous work [14]. We assumed the ECM consisted of collagen (volume fraction, v/v = 14%, fiber radius = 20 nm) and GAG (v/v = 0.078%, fiber radius = 0.55 nm) while the basement membrane had an effective fiber composition with v/v = 15.4%, fiber radius = 0.70 nm taken from experimental fitting of the hydraulic conductivity of Matrigel [15]. We first estimated the aqueous diffusivity of VEGF and protease, D_aq_, using a MW to diffusivity correlation at 23°C [16], and then using the Stokes-Einstein relation, estimated the solute radius, r_s_, and aqueous diffusivities at 37 °C (based on water viscosities, μ_23°C_ 0.93 cP, μ_37°C_ = 0.69 cP [17]). We then corrected for the diffusion hindrance presented by the soluble protein content of the interstitial fluid and the interstitial matrix fibers. We can estimate the former at ~24% based on a protein content of interstitial fluid of 20.6 g/L [18] using linear interpolation between water and blood plasma (protein ~70 g/L, μ_37°C_ = 1.26 cP), resulting in μ_37°C_ = 0.86 cP. The effects of matrix fibers can be approximated using Ogston’s relation [19]:

$$\alpha=\theta\left(1+\frac{r_{s}}{r_{f}}\right)^{2} (SV-1)$$

$$D=D_{IS}\cdot\exp\left(-\alpha^{1/2}\right)=D_{IS}\cdot\exp\left(-(1+\frac{r_{s}}{r_{f}})\theta^{1/2}\right) (SV-2)$$

where r_s_ is the solute radius, r_f_ the fiber radius, θ the fiber volume fraction in the interstitial space, and D_IS_ is the diffusivity of the solute in the interstitial solution. This form of the Ogston’s equation is different than that used in previous studies [14,20] and can partly explain the observed overestimation of the diffusivity using the form of the Ogston relation found in Johnson et al. [20] (*results not shown*). To account for both collagen and GAG in the ECM, we can formulate the diffusivity as

$$D=D_{aq}\cdot\exp\left(-(\alpha_{Collagen}+\alpha_{GAG})^{1/2}\right) (SV-3)$$

which is also different than used previously [14]. For VEGF_165_ (45 kDa), r_s_ is estimated at 2.468 nm with D_aq_ (37°C) = 133 μm^2^/s. Taking into account the increased viscosity of interstitial fluid due to soluble proteins, D_IS_ = 107 μm^2^/s. α_Collagen_ = 0.18 and α_GAG_ = 0.024, resulting in a final D = 68.6 μm^2^/s. (We should note that this form of the Ogston’s relation correctly recapitulates the increased effect of collagen over GAG in the hindrance of biological matrices [21], while the previous formulation does not). Similarly, using the basement membrane properties given above, we can derive D = 18.0 μm^2^/s. The calculated diffusivity of various VEGF forms, bFGF, and proteases at 37°C is provided below in Table S2.

Previously, the diffusivity of bFGF (18 kDa) in the Descemet’s basement membrane has been measured at D ~ 0.7 μm^2^/s in an aqueous PBS-BSA (1 mg/mL) solution at 4°C. Using Ogston’s relation and the basement membrane properties found above for Matrigel, we calculate a value that is ~25 fold larger (~17 μm^2^/s at 4°C). This situation can possibly be remedied using alternate models of matrix diffusive hindrance (e.g. using combined hydrodynamic and obstruction models, i.e. combining the Johansson and Effective-Medium models yielding ~5 μm^2^/s [20], or combining the Johansson and Clague-Philips models yielding ~4 μm^2^/s [19]), which are significant improvements over the Ogston model in this case. However, uncertainties in the composition of various matrices and the limited accuracy of diffusion models in the dense but thin fiber regime (e.g. where θ is large but r_f_ is small as it is for basement membranes) likely makes further analysis difficult. (For comparison, α_BM_ (45kDa) = 3.2 is much larger than α_ECM_ (45kDa) = 0.2 even though the fiber volume in both matrices is similar.) Fortunately, the basement membrane diffusivity does not significantly affect our results unless it is made extremely small, D ~ 10^−2^ μm^2^/s.

Table S2. Diffusivity in Matrices

| **Solute** | **kDa** | **ECM** | **BM** |
| --- | --- | --- | --- |
| VEGF189-189 | 52 | 65.0 | 16.0 |
| VEGF165-165† | 45 | 68.6 | 18.0 |
| VEGF165-114 | 39 | 72.3 | 20.2 |
| VEGF114-114 | 32 | 77.8 | 23.6 |
| VEGFc‡ | 6 | 141.7 | 71.3 |
| Plasmin | 86 | 53.9 | 10.3 |
| MMP3 (active) | 45 | 68.9 | 18.0 |
| MMP9 (active) | 82 | 54.8 | 10.7 |
| bFGF | 18 | 95.8 | 35.7 |

Legend:

Values calculated using Ogston’s diffusion model assuming matrix properties given in *above*, for 37°C and assuming interstitial fluid protein content of 21 g/L. All values expressed in μm^2^/s.

† Diffusivities of all VEGF isoforms in the simulations were assumed equal to that of VEGF_165-165_.

‡ Assuming globular configuration.

**Section SVI. Analytical expressions for VEGF transport and cell-surface proteolysis**

**SVI.1 Effective rate constants for VEGF_165_ cell-surface proteolysis**

Cell-surface proteases are thought to be important in the pericellular proteolysis of the ECM and growth factors [3,22-24]. In Fig. 4 of the manuscript, we show that conversion of VEGF_165_ by cell-surface proteases is not feasible at physiological protease levels, while conversion of cell surface or basement membrane VEGF_165_-HSPG are likely to be very strong. To analyze this behavior, we derived effective rate constants of proteolysis by a single cell, similar to that typically used to describe receptor-ligand association [25]. We consider a spherical cell endowed with cell-surface activity (VEGF cleaving proteases, receptors for VEGF, and HSPG) in an infinite volume (Fig. S3A). In this analysis, we neglect the diffusive hindrance of the basement membrane layer (as D_BM_/d_BM_ >> D_ECM_/R_spout_). At steady state, HSPGs do not contribute to VEGF transport in the extracellular matrix volume (due to the absence of ECM proteases in this analysis). As a result, VEGF transport (specifically, the interstitial-fluid concentration of VEGF_165_) in this volume is governed by the differential equation

$$D\frac{1}{r^{2}}\frac{\partial}{\partial r}\left(r^{2}\frac{\partial[V_{165}]}{\partial r}\right)=0 (SVI.1-1)$$

with a far-field boundary condition, V(r=∞) = V_0_, where V_0_ is the VEGF concentration found in the ECM’s available pores. At the cell surface, the rate of reaction is equal to the diffusive flux, i.e.

$$\begin{matrix} 4\pi R_{Eff}^{2}K_{ECM}D\left.\frac{\partial[V_{165}]}{\partial r}\right|_{r=\mathrm{Re}ff}=k_{on}^{165,R2}\left\{R2\right\}[V_{165}]-k_{off}^{165,R2}\left\{V_{165}R2\right\}+k{}_{P}\left\{P\right\}[V_{165}]+k_{on}^{165,H}\left\{H\right\}[V_{165}]-k_{off}^{165,H}\left\{V_{165}H\right\} \\ (SVI.1-2) \end{matrix}$$

Here the {} brackets, e.g. {R2}, {V_165_R2}, {P}, {H}, and {V_165_H}, denote the number of molecules of each of the respective species present at the cell surface and basement membrane volume. We will use this convention in the rest of this section. R_eff_ is the effective radius of a spherical cell with equal surface area as our model tip cell. Note that the above equation is a simplification that uses K_ECM_ instead of explicitly representing K_BM_ and the basement membrane layer thickness, which is valid because of the basement membrane layer’s negligible effect on the overall VEGF diffusion. The species {V_165_R2}, {R2}, {V_165_H}, {H}, and {P} are governed by the following equations at steady state:

$$k_{on}^{165,R2}\left\{R2\right\}[V_{165}]=k_{off}^{165,R2}\left\{V_{165}R2\right\}+k_{\mathrm{int}}\left\{V_{165}R2\right\}$$

$$k_{on}^{165,H}\left\{H\right\}[V_{165}]=k_{off}^{165,H}\left\{V_{165}H\right\}+k_{P}[P]\left\{V_{165}H\right\}$$

where [P] is the interstitial-fluid concentration of {P} proteases in the available volume of the basement membrane. As a result, at steady state, the VEGF_165_ boundary condition can be reduced to:

$$4\pi R_{Eff}^{2}K_{ECM}D\left.\frac{\partial[V_{165}]}{\partial r}\right|_{r=\mathrm{Re}ff}=k_{\mathrm{int}}\left\{V_{165}R2\right\}+k{}_{P}\left\{P\right\}[V_{165}]+k_{P}[P]\left\{V_{165}H\right\} (SVI.1-3)$$

Given that {R2} = {R2}_Total_ – {V_165_R2} and {H} = {H}_Total_ – {V_165_H}, we can solve for {V_165_R2} and {V_165_H} at the cell surface as:

$$\left\{V_{165}R2\right\}=\frac{\left\{R2\right\}_{Total}k_{on}^{165,R2}[V_{165}]}{k_{off}^{165,R2}+k_{\mathrm{int}}+k_{on}^{165,R2}[V_{165}]}$$

$$\left\{V_{165}H\right\}=\frac{\left\{H\right\}_{Total}k_{on}^{165,H}[V_{165}]}{k_{off}^{165,H}+k_{P}[P]+k_{on}^{165,H}[V_{165}]}$$

We are interested in the low VEGF limit, i.e. low Autocrine numbers [26], since interstitial VEGF levels are small [27] and because it will represent an upper bound in the degree of VEGF_165_ conversion (or the receptor-mediated capture probability). As a result, the VEGF_165_ boundary condition becomes:

$$4\pi R_{Eff}^{2}K_{ECM}D\left.\frac{\partial[V_{165}]}{\partial r}\right|_{r=\mathrm{Re}ff}=\left(k{}_{P}\left\{P\right\}+\frac{k_{\mathrm{int}}\cdot\left\{R2\right\}_{Total}k_{on}^{165,R2}}{k_{off}^{165,R2}+k_{\mathrm{int}}}+\frac{k_{P}[P]\cdot\left\{H\right\}_{Total}k_{on}^{165,H}}{k_{off}^{165,H}+k_{P}[P]}\right)\cdot[V_{165}] (SVI.1-4)$$

Solving the VEGF_165_ transport equation with its associated boundary conditions, and setting

$$4\pi R_{eff}^{2}K_{ECM}D\left.\frac{\partial[V_{165}]}{\partial r}\right|_{r=\mathrm{Re}ff}=k_{f,overall}V_{0}$$

where k_f,overall_ is defined to be the interstitial-fluid rate constant describing the overall VEGF_165_ transport into cell, gives us,

$$\text{k}_{\text{f, overall}}=\frac{4\pi DR_{eff}K_{ECM}\cdot\left(k_{P}\left\{P\right\}+k_{on}\frac{k_{P}[P]}{k_{P}[P]+k_{off}}\left\{H\right\}_{Total}+k_{on}\frac{k_{\mathrm{int}}}{k_{\mathrm{int}}+k_{off}}\left\{R2\right\}_{Total}\right)}{4\pi DR_{eff}K_{ECM}+k_{P}\left\{P\right\}+k_{on}\frac{k_{P}[P]}{k_{P}[P]+k_{off}}\left\{H\right\}_{Total}+k_{on}\frac{k_{\mathrm{int}}}{k_{\mathrm{int}}+k_{off}}\left\{R2\right\}_{Total}} (SVI.1-5)$$

This can be expressed more symbolically as,

$$\text{k}_{\text{f, overall}}=\frac{k_{+}\cdot\left(k_{VP}+k_{VHP}+k_{VR}\right)}{k_{+}+k_{VP}+k_{VHP}+k_{VR}}$$

where:

$$k_{+}=4\pi DR_{eff}K_{ECM}$$

$$k_{VP}=k_{P}\left\{P\right\}$$

$$k_{VHP}=k_{on}\frac{k_{P}[P]}{k_{P}[P]+k_{off}}\left\{H\right\}_{Total}$$

$$k_{VR}=k_{on}\frac{k_{\mathrm{int}}}{k_{\mathrm{int}}+k_{off}}\left\{R\right\}_{Total}$$

We are interested in the interstitial-fluid, cell-surface VEGF_165_ concentration, which can be calculated as:

$$[V_{165}]=V_{0}\cdot\left(1-\frac{k_{f,overall}}{k_{+}}\right) (SVI.1-6)$$

This represents the cell-surface VEGF_165_ concentration, depleted from its far-field value V_0_ due to either proteolytic conversion or VEGFR-mediated internalization. The concentration of VEGF_165_ bound to HSPGs (for low free VEGF levels) at the cell surface is then given by:

$$[V_{165}H]=\frac{k_{on}^{165,H}[V_{165}][H]_{Total}}{k_{off}^{165,H}+k_{P}[P]+k_{on}^{165,H}[V_{165}]}\approx\frac{k_{on}^{165,H}[V_{165}][H]_{Total}}{k_{off}^{165,H}+k_{P}[P]} (SVI.1-7)$$

In our model, the sprout is approximated as a cylinder; thus the effective radius of a spherical cell of equivalent surface area as our tip cell of length, L_tip_, becomes R_eff_ = 0.5∙(2∙R_sprout_∙L_tip_ + R_sprout_^2^)^1/2^ = 6.4 μm. Given a basement membrane thickness of 43 nm and a K_BM_ of 0.2 (see manuscript, *Methods, Geometrical and Transport Parameters*), 10^5^ proteases translate to an interstitial-fluid concentration of 37.5 μM.

The depletion (the net contribution of internalization and proteolysis) of VEGF_165_ and VEGF_165_-HSPG at the cell surface can be expressed as:

$$depletion_{165}=\frac{k_{f,overall}}{k_{+}}=1-\left(1-\frac{k_{VP}+k_{VHP}+k_{VR}}{k_{+}+k_{VP}+k_{VHP}+k_{VR}}\right) (SVI.1-8)$$

$$depletion_{165-HSPG}=1-\frac{[V_{165}H]_{\Pr otease}}{[V_{165}H]_{No \Pr otease}}=1-\left(1-\frac{k_{P}[P]}{k_{off}^{165,H}+k_{P}[P]}\right)\left(1-\frac{k_{VP}+k_{VHP}+k_{VR}}{k_{+}+k_{VP}+k_{VHP}+k_{VR}}\right) (SVI.1-9)$$

This formulation indicates that the depletions of VEGF_165_ and VEGF_165_-HSPG can be expressed in similar forms. Notably, similar to how VEGF_165_ is replenished by the diffusion-limit rate, k_+_, the replenish-rate of VEGF_165_-HSPG is given by k_off_^165,H^ (for negligible VEGF_165_ depletion), i.e. the rate at which another VEGF molecule can bind to HSPG at steady state. This translates into a molar dissociation rate constant of k_off,Molar_^165,H^ = k_off_^165,H^ ∙N_av_∙Ω_Tip BM_ ~ 2.67∙10^7^ M^−1^s^−1^, where N_av_ is the Avogadro’s number and Ω_Tip BM_ is the total available volume of the basement membrane region.

We should note that the above analysis is only valid for a single spherical cell in isolation. In our sprout model, we assume that the stalk cells also express VEGFR2, and as a result, the overall VEGF depletion due to the entire sprout internalizing VEGF is greater than that predicted by these equations. Another point is that reactions occurring between molecules on the cell surface can often be diffusion limited due to slow cell-surface diffusivities (D ~ 10^−2^ μm^2^/s [25]). We treat the reaction between cell-surface protease and pericellular/cell-surface HSPGs as being reaction limited due to the large value of K_m_ between VEGF_165_ and plasmin (see *Supplement, Section S1*), which indicates that VEGF proteolysis is association limited.

Next, we apply these results to our model of the cell-surface proteolysis of VEGF. For our model, VEGF_165_ diffusion to the tip cell surface is very fast, characterized by the rate constant, k_+_ = 2.83∙10^12^ M^−1^s^−1^. At {P} = 10^5^ and {R2} = 10^4^, proteolysis of the soluble VEGF occurs at k_VP_ = 6.31∙10^7^ M^−1^s^−1^, proteolysis through HSPG binding at k_VHP_ = 1.02∙10^10^ M^−1^s^−1^, and internalization at k_VR_ = 2.19∙10^10^ M^−1^s^−1^. k_f,overall_ has a value of 3.18∙10^10^ M^−1^s^−1^, indicating that VEGF depletion at the cell surface is largely reaction limited (VEGF_165_ depletion of 1.13%). A majority of this depletion occurs due to capture by VEGFR2 (probability of capture = k_VR_/k_+_), resulting in a receptor-mediated depletion of 0.8%. We note that our theoretical estimates were usually within 5% of computationally predicted values in Fig. 4 of manuscript.

The dependence of the VEGF_165_ depletion on cell-surface protease levels is shown in Fig. S3B. Note that the depletion of VEGF_165_-HSPG is apparent at ~5-decade lower cell-surface protease levels than that of VEGF_165_, which requires ~10^10^ proteases. The 5-decade difference is explained by the rates at which each of the species is replenished. For VEGF_165_, this is k_+_ (or k_+_/(N_av_∙ Ω_Tip BM_) = 1059 s^-1^) while for VEGF_165_-HSPG, it is k_off_^165,HSPG^ = 0.01 s^−1^ (equivalent to a volumetric rate of 2.67∙10^7^ M^−1^s^−1^). As a final point, we also note that while HSPGs potentiate VEGF_165_ cleavage, their effect is limited by the rate of association, resulting in a maximal depletion of 1.27% at 2.6 μM HSPG.


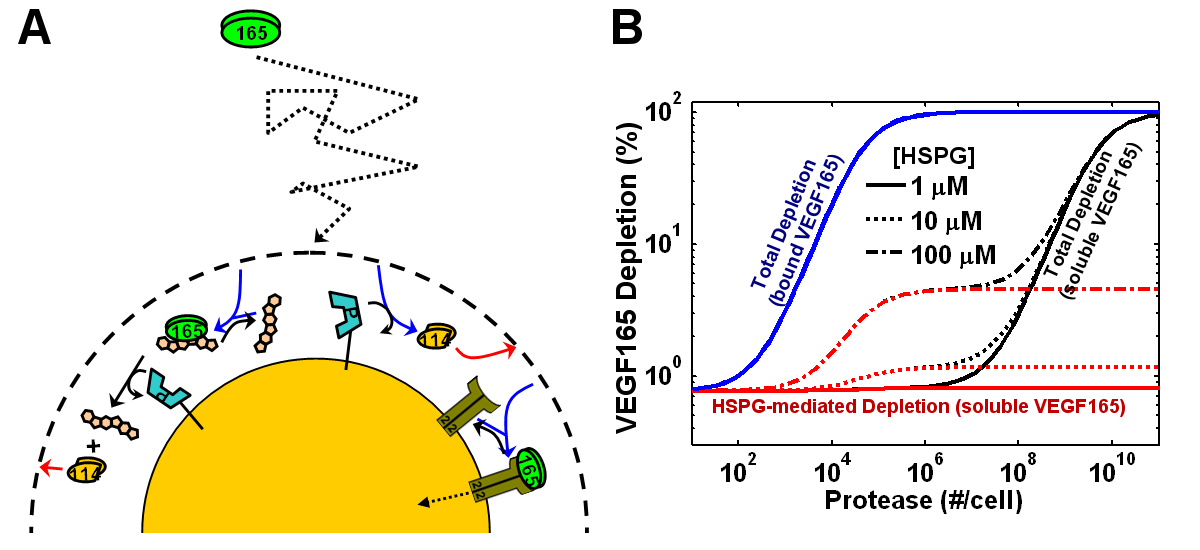


Figure S3 – Cell-surface mediated VEGF proteolysis. Proteases were restricted to the basement membrane layer surrounding the tip cell (A). VEGF_165_ cleavage can occur either via direct encounter with a protease or after an initial complexation with HSPG. B, analytical results for diffusion-limited VEGF proteolysis at the cell surface. Depletion reflects the total effect of VEGF_165_ removal by proteolysis (i.e. conversion) and internalization. VEGF_165_-HSPG depletion (blue) is independent of [HSPG] (overlapped lines). Soluble VEGF_165_ depletion mediated through proteolysis of both soluble and HSPG-bound forms (black), while in red, VEGF proteolysis occurs only while bound to HSPGs. Non-zero depletion in at any protease levels (~0.8%) is due to depletion by VEGFR2-mediated internalization.

**SVI.2 Kinetics of VEGF clearance and VEGF conversion**

Due to the numerous processes involved (secretion, clearance, HSPG binding, and proteolysis), it would be useful to understand what factors determine the overall degree of proteolysis in the system, as well as the rate at which the processes proceed to reach steady state. We assume well-stirred behavior and derive ordinary differential equations describing the VEGF dynamics in the available volume of the system. There are three primary uncleaved VEGF compartments: soluble VEGF, matrix-bound VEGF, and receptor-bound VEGF. Since we look at the uncleaved fraction of VEGF, and since total soluble VEGF will be constant given identical rates constants of internalization and clearance for VEGF_165_ and VEGF_114_, we can disregard the cleaved VEGF in this analysis.

$$\begin{matrix} \frac{d\text{[V}_{165}\text{]}}{d\text{t}}=q_{V}\frac{A_{s}}{\Omega}-k_{clear}\text{[V}_{165}\text{]}\frac{A_{s}}{\Omega}+\left(k_{off}^{V,R2}[V_{165}R2]-k_{on}^{V,R2}[V_{165}][R2]\right)\frac{A_{v}}{\Omega} \\ +k_{off}^{V,H}[V_{165}H]-k_{on}^{V,H}[V_{165}][H]-k_{P}[P][V_{165}] (SVI.2-1) \end{matrix}$$

$$\frac{d\text{[V}_{165}\text{H]}}{d\text{t}}=k_{on}^{V,H}[V_{165}][H]-k_{off}^{V,H}[V_{165}H]-k_{P}[P][V_{165}H] (SVI.2-2)$$

$$\frac{d\text{[V}_{165}\text{R2]}}{d\text{t}}=k_{on}^{V,R2}[V_{165}][R2]-k_{off}^{V,R2}[V_{165}R2]-k_{\mathrm{int}}^{VR2}[V_{165}R2] (SVI.2-3)$$

where A_s_ is the available area of z-plane edge of the domain volume, i.e. π∙R_edge_^2^∙K_ECM_, or 6.68∙10^3^ μm^2^, A_v_ is the vessel surface area (1018 μm^2^), and Ω is the total available volume in the domain, or 1.07∙10^6^ μm^3^. Note that the definition of Ω differs from that used in the previous section.

As a simplification, we assume that all reversible reactions are at a pseudo-steady-state. This is justified since these reactions will equilibrate very rapidly. HSPG binding, for example, has effective association and dissociation rates of k_on_[H] ~ 4.2∙10^5^ M^−1^s^−1^ ∙ 750∙10^−9^ M = 0.315 s^−1^ and k_off_ = 0.01 s^−1^, respectively, giving an overall rate of 0.325 s^-1^ (or τ^165,H^ = 3.08 s). Receptor binding is only slightly slower: effective association rate = k_on_^V,R2^[R2]_Total_∙A_v_/Ω = 3.07∙10^-4^ s^-1^, dissociation rate = k_off_ = 0.001 s^-1^, total rate = 0.00131 s^-1^ (τ^165,R2^ = 0.21 h). This method to calculate the time constant of reversible reactions is different than previous estimates where only the dissociation or association rates individually are taken into account [14,28]. We have verified our approach for the accurate estimation of kinetics over a large range of concentration and kinetic parameters (*not shown*).

Note that we can add equations SVI.2-1-3 to yield an equation for total VEGF, for which we are interested in determining the dynamics:

$$\begin{matrix} \frac{d\text{[V}_{165}\text{]}}{d\text{t}}+\frac{d\text{[V}_{165}\text{H]}}{d\text{t}}+\frac{d\text{[V}_{165}\text{R2]}}{d\text{t}}=q_{V}\frac{A_{s}}{\Omega}-k_{clear}\text{[V}_{165}\text{]}\frac{A_{s}}{\Omega}-k_{\mathrm{int}}^{VR2}[V_{165}R2]\cdot\frac{A_{v}}{\Omega} \\ -k_{P}[P][V_{165}H]-k_{P}[P][V_{165}] (SVI.2-4) \end{matrix}$$

We approximate HSPG-bound VEGF as [V_165_H] = k_on_^V,H^∙[H]_Total_∙[V_165_]/(k_off_^V,H^ + k_P_∙[P]) and receptor-bound VEGF as k_on_^V,R2^∙[R2]_Total_∙[V_165_]/(k_off_^V,R2^ + k_int_^V,R2^) in the limit of low free VEGF, i.e. [V_165_] << K_d_^V,H^ and K_d_^V,R2^. Substituting these relations, we get

$\begin{matrix} \frac{d\text{[V}_{165}\text{]}}{d\text{t}}\cdot\left(1+\frac{k_{on}^{V,H}\cdot[H]_{Total}}{k_{off}^{V,R2}+k_{P}[P]}+\frac{k_{on}^{V,R2}\cdot[R2]_{Total}}{k_{off}^{V,R2}+k_{\mathrm{int}}^{VR2}}\right) \\ =q_{VEGF}\frac{A_{s}}{\Omega}-\left(k_{clear}\cdot\frac{A_{s}}{\Omega}+k_{\mathrm{int}}^{VR2}\frac{k_{on}^{V,R2}\cdot[R2]_{Total}}{k_{off}^{V,R2}+k_{\mathrm{int}}^{VR2}}\cdot\frac{A_{v}}{\Omega}+k_{P}[P]\cdot\left(1+\frac{k_{on}^{V,H}\cdot[H]_{Total}}{k_{off}^{V,R2}+k_{P}[P]}\right)\right)\cdot\text{[V}_{165}\text{]} (SVI.2-5) \end{matrix}$This equation is very informative. The multiplicative factor to d[V_165_]/dt details where VEGF is stored, i.e. in solution, bound to HSPGs, and bound to VEGF receptors. The terms within this factor detail how much is relatively stored in each compartment. The right side of the equation details all of the VEGF_165_ fluxes, including VEGF secretion and the processes that remove VEGF_165_ from the system, e.g. clearance, receptor-mediated endocytosis, and proteolysis. This equation is a first order differential equation where the effective rate constant is given by the net rate of VEGF_165_ removal from the system, divided by the degree of VEGF storage.

$$k'=\frac{\left(k_{clear}\cdot\frac{A_{s}}{\Omega}+k_{\mathrm{int}}^{VR2}\frac{k_{on}^{V,R2}\cdot[R2]_{Total}}{k_{off}^{V,R2}+k_{\mathrm{int}}^{VR2}}\cdot\frac{A_{v}}{\Omega}+k_{P}[P]\cdot\left(1+\frac{k_{on}^{V,H}\cdot[H]_{Total}}{k_{off}^{V,R2}+k_{P}[P]}\right)\right)}{\left(1+\frac{k_{on}^{V,H}\cdot[H]_{Total}}{k_{off}^{V,R2}+k_{P}[P]}+\frac{k_{on}^{V,R2}\cdot[R2]_{Total}}{k_{off}^{V,R2}+k_{\mathrm{int}}^{VR2}}\right)} (SVI.2-6)$$

In the numerator, clearance occurs at a rate λ_C_ ≡ k_clear_∙A_s_/Ω ~ 5.42∙10^−4^ s^−1^; internalization at λ_I_ = 6.72∙10^−5^ s^−1^; while for [P] = 10 nM, proteolysis occurs at λ_P_ = 2.04∙10^-4^ s^-1^.The total storage contributions in the denominator are 1 for the soluble fraction, 31.5 for HSPG-bound VEGF, and 0.24 for VEGFR2-bound VEGF. The effective rate constant of the system is equated to k’ = 2.49∙10^-5^ s^-1^, giving an overall time constant of τ = 1/k’ ~ 11.2 h), which agrees well with the time constant observed in the computational model of 11.9 h (*not shown*).

Knowledge of these rates also allows us to calculate the expected VEGF conversion for different protease concentrations. The steady-state soluble VEGF_165_ conversion can be approximated as

$$1-\frac{[VEGF_{165}]_{\Pr otease}}{[VEGF_{165}]_{No\Pr otease}}=\frac{\lambda_{P}}{\lambda_{P}+\lambda_{C}+\lambda_{I}} (SVI.2-7)$$

where λ_P_, λ_C_, and λ_I_, are the proteolysis, clearance, and internalization rates, respectively, given in equation SVI.2-6.. Proteolysis results in a conversion of 25%, which agrees well with the computationally observed value of 27%.

In our model of the sprout, the storage contribution of the receptors is very small compared to that of the HSPGs and we can neglect it for simplicity. This leads to a time constant

$$\tau=\frac{1}{k'}=\left(k_{clear}\cdot\frac{A_{s}}{\Omega}\cdot\frac{K_{d}^{V,H}}{[H]_{Total}+K_{d}^{V,H}}+k_{\mathrm{int}}^{VR2}\cdot\frac{k_{on}^{V,R2}\cdot[R2]_{Total}}{k_{off}^{V,R2}+k_{\mathrm{int}}^{VR2}}\cdot\frac{A_{v}}{\Omega}\cdot\frac{K_{d}^{V,H}}{[H]_{Total}+K_{d}^{V,H}}+k_{P}[P]\right)^{-1} (SVI.2-8)$$

Recognizably, VEGF clearance and internalization is hampered by HSPG binding to a factor of K_d_^V,H^/(K_d_^V,H^ + [H]_Total_) (or an increase in the residence time by a factor 1+[H]_Total_/K_d_^165,H^). Note that the rate of proteolysis is not influenced by HSPGs because VEGF proteolysis can occur regardless of whether VEGF is bound to HSPGs or not (i.e. HSPGs do not protect VEGF against proteases). If we had instead assumed that HSPGs protect VEGF against proteolysis, the proteolysis rate would also be lowered by a factor of K_d_^165,H^/([H]_Total_ + K_d_^165,H^), similar to that of clearance and internalization. The ratio Ω/A has a unit of length and determines the effective length that VEGF must travel through the tissue while becoming proteolyzed. Longer effective lengths, similar to HSPGs, thus elicit greater VEGF conversion due to the increased residence time of VEGF in the tissue.

**References**

1. Keyt BA, Berleau LT, Nguyen HV, Chen H, Heinsohn H, et al. (1996) The carboxyl-terminal domain (111-165) of vascular endothelial growth factor is critical for its mitogenic potency. J Biol Chem 271: 7788-7795.

2. Sires UI, Griffin GL, Broekelmann TJ, Mecham RP, Murphy G, et al. (1993) Degradation of entactin by matrix metalloproteinases. Susceptibility to matrilysin and identification of cleavage sites. J Biol Chem 268: 2069-2074.

3. Lee S, Jilani SM, Nikolova GV, Carpizo D, Iruela-Arispe ML (2005) Processing of VEGF-A by matrix metalloproteinases regulates bioavailability and vascular patterning in tumors. J Cell Biol 169: 681-691.

4. Ramos-DeSimone N, Hahn-Dantona E, Sipley J, Nagase H, French DL, et al. (1999) Activation of matrix metalloproteinase-9 (MMP-9) via a converging plasmin/stromelysin-1 cascade enhances tumor cell invasion. J Biol Chem 274: 13066-13076.

5. Yao C, Roderfeld M, Rath T, Roeb E, Bernhagen J, et al. (2006) The impact of proteinase-induced matrix degradation on the release of VEGF from heparinized collagen matrices. Biomaterials 27: 1608-1616.

6. Imai K, Hiramatsu A, Fukushima D, Pierschbacher MD, Okada Y (1997) Degradation of decorin by matrix metalloproteinases: identification of the cleavage sites, kinetic analyses and transforming growth factor-beta1 release. Biochem J 322 ( Pt 3): 809-814.

7. Tzafriri AR, Bercovier M, Parnas H (2002) Reaction diffusion model of the enzymatic erosion of insoluble fibrillar matrices. Biophys J 83: 776-793.

8. Welgus HG, Jeffrey JJ, Eisen AZ (1981) The collagen substrate specificity of human skin fibroblast collagenase. J Biol Chem 256: 9511-9515.

9. Aimes RT, Quigley JP (1995) Matrix metalloproteinase-2 is an interstitial collagenase. Inhibitor-free enzyme catalyzes the cleavage of collagen fibrils and soluble native type I collagen generating the specific 3/4- and 1/4-length fragments. J Biol Chem 270: 5872-5876.

10. Seltzer JL, Weingarten H, Akers KT, Eschbach ML, Grant GA, et al. (1989) Cleavage specificity of type IV collagenase (gelatinase) from human skin. Use of synthetic peptides as model substrates. J Biol Chem 264: 19583-19586.

11. Welgus HG, Burgeson RE, Wootton JA, Minor RR, Fliszar C, et al. (1985) Degradation of monomeric and fibrillar type III collagens by human skin collagenase. Kinetic constants using different animal substrates. J Biol Chem 260: 1052-1059.

12. Lauer-Fields JL, Sritharan T, Stack MS, Nagase H, Fields GB (2003) Selective hydrolysis of triple-helical substrates by matrix metalloproteinase-2 and -9. J Biol Chem 278: 18140-18145.

13. Mac Gabhann F, Popel AS (2007) Interactions of VEGF isoforms with VEGFR-1, VEGFR-2, and neuropilin in vivo: a computational model of human skeletal muscle. Am J Physiol Heart Circ Physiol 292: H459-474.

14. Filion RJ, Popel AS (2005) Intracoronary administration of FGF-2: a computational model of myocardial deposition and retention. Am J Physiol Heart Circ Physiol 288: H263-279.

15. Katz MA, Barrette T, Krasovich M (1992) Hydraulic conductivity of basement membrane with computed values for fiber radius and void volume ratio. Am J Physiol 263: H1417-1421.

16. Berk DA, Yuan F, Leunig M, Jain RK (1993) Fluorescence photobleaching with spatial Fourier analysis: measurement of diffusion in light-scattering media. Biophys J 65: 2428-2436.

17. Weast RC, Astle MJ, Beyer WH (1986) Handbook of Chemistry and Physics. Boca Raton, Florida: CRC Press, Inc.

18. Fogh-Andersen N, Altura BM, Altura BT, Siggaard-Andersen O (1995) Composition of interstitial fluid. Clin Chem 41: 1522-1525.

19. Amsden B (1998) Solute Diffusion within Hydrogels. Mechanisms and Models. Macromolecules 31: 8382-8395.

20. Johnson EM, Berk DA, Jain RK, Deen WM (1996) Hindered diffusion in agarose gels: test of effective medium model. Biophys J 70: 1017-1023.

21. Levick JR (1987) Flow through interstitium and other fibrous matrices. Q J Exp Physiol 72: 409-437.

22. Yu Q, Stamenkovic I (1999) Localization of matrix metalloproteinase 9 to the cell surface provides a mechanism for CD44-mediated tumor invasion. Genes Dev 13: 35-48.

23. Yu Q, Stamenkovic I (2000) Cell surface-localized matrix metalloproteinase-9 proteolytically activates TGF-beta and promotes tumor invasion and angiogenesis. Genes Dev 14: 163-176.

24. Karagiannis ED, Popel AS (2006) Distinct modes of collagen type I proteolysis by matrix metalloproteinase (MMP) 2 and membrane type I MMP during the migration of a tip endothelial cell: insights from a computational model. J Theor Biol 238: 124-145.

25. Lauffenburger DA, Linderman JJ (1993) Receptors: Models for Binding, Trafficking, and Signaling. Receptors. New York: Oxford University Press, Inc. pp. 144-151.

26. Shvartsman SY, Wiley HS, Deen WM, Lauffenburger DA (2001) Spatial range of autocrine signaling: modeling and computational analysis. Biophys J 81: 1854-1867.

27. Kut C, Mac Gabhann F, Popel AS (2007) Where is VEGF in the body? A meta-analysis of VEGF distribution in cancer. Br J Cancer 97: 978-985.

28. Dowd CJ, Cooney CL, Nugent MA (1999) Heparan sulfate mediates bFGF transport through basement membrane by diffusion with rapid reversible binding. J Biol Chem 274: 5236-5244.
